# Admission risk factors and predictors of moderate or severe pediatric acute pancreatitis: A systematic review and meta-analysis

**DOI:** 10.3389/fped.2022.947545

**Published:** 2022-09-30

**Authors:** Márk Félix Juhász, Zoltán Sipos, Klementina Ocskay, Péter Hegyi, Anikó Nagy, Andrea Párniczky

**Affiliations:** ^1^Heim Pál National Pediatric Institute, Budapest, Hungary; ^2^Medical School, Institute for Translational Medicine, University of Pécs, Pécs, Hungary; ^3^Centre for Translational Medicine, Department of Medicine, University of Szeged, Szeged, Hungary; ^4^Division of Translational Medicine, First Department of Medicine, Medical School, University of Pécs, Pécs, Hungary; ^5^Centre for Translational Medicine, Semmelweis University, Budapest, Hungary

**Keywords:** pediatric pancreatitis, severity, predictive factors, on-admission, meta-analysis

## Abstract

**Introduction:**

Pediatric acute pancreatitis (PAP) has an increasing incidence and is now estimated to be almost as common as in adults. Up to 30% of patients with PAP will develop moderate or severe disease course (M/SPAP), characterized by organ failure, local or systemic complications. There is still no consensus regarding on-admission severity prediction in these patients. Our aim was to conduct a systematic review and meta-analysis of available predictive score systems and parameters, and differences between on-admission parameters in mild and M/SPAP.

**Methods:**

We conducted a systematic search on the 14^th^ February, 2022 in MEDLINE, Embase and CENTRAL. We performed random-effects meta-analysis of on-admission differences between mild and M/SPAP in laboratory parameters, etiology, demographic factors, etc. calculating risk ratios (RR) or mean differences (MD) with 95% confidence intervals (CI) and created forest plots. For the meta-analysis of predictive score systems, we generated hierarchical summary receiver operating characteristic curves using a bivariate model. Chi-squared tests were performed and I^2^ values calculated to assess statistical heterogeneity.

**Results:**

We included 44 studies – mostly retrospective cohorts – in our review. Among predictive score systems examined by at least 5 studies, the modified Glasgow scale had the highest specificity (91.5% for values ≥3), and the Pediatric Acute Pancreatitis Severity score the highest sensitivity (63.1% for values ≥3). The performance of other proposed score systems and values were summarized. Traumatic (RR: 1.70 95% CI: 1.09–2.67) and drug–induced (RR: 1.33 95% CI: 0.98–1.87) etiologies were associated with a higher rate of M/SPAP, while anatomical (RR: 0.6195% CI: 0.38–0.96) and biliary (RR: 0.72 95% CI: 0.53–0.99) PAP tended to be less severe.

**Discussion:**

Many predictive score systems were proposed to assess the possibility of M/SPAP course. The most commonly used ones exhibit good specificity, but subpar sensitivity. Our systematic review provides a rigorous overview of predictive options assessed thus far, that can serve as a basis for future improvement of scores *via* the addition of parameters with a better observed sensitivity: e.g., lipase exceeding 7-times the upper threshold, hemoglobin, etc. The addition of etiological factors is another possibility, as they can herald a more severe disease course.

**Systematic review registration:**

https://www.crd.york.ac.uk/prospero/display_record.php?RecordID=307271, PROSPERO, identifier: CRD42022307271.

## Introduction

While in the adult emergency department, acute pancreatitis is a common differential diagnostic concern (1), pediatric acute pancreatitis (PAP) is a less frequently sought diagnosis, mostly because it was for long regarded as a rarely presenting disorder. On the contrary, the last few decades' publications report its increasing incidence, now estimated to be 3-13/100,000/year, which approaches the 13-45/100,000/year incidence seen in adults (2–7). While this trend might reflect a true increase in incidence, there is no doubt that the increasing diagnostic awareness (pancreatic enzyme measurement) greatly contributes (5, 8). Either way, more and more patients with PAP are discovered and hospitalized in need of adequate treatment.

As of yet, however, there are no known specific therapeutic options in PAP. The management of these patients is based on pain control, intravenous fluid replacement, adequate nutrition, monitoring complications and intensive care if necessary (9). Fortunately, as opposed to adults, where 15–30% of patients have a moderate disease course and 10–20% severe, with up to 40% mortality (10–12), PAP usually has a more benign course, with only 20–30% of cases being classified as moderate or severe (M/SPAP) in the majority of pediatric studies (see Table 1). Thus, only around every fourth or fifth pediatric patient will develop local complications, even less organ failure. But the low number of M/SPAP (especially together with the lower diagnostic awareness still persisting in many centers) can lead to the delayed recognition of these children. Great emphasis should be placed on their early identification, in order for a prompt response and transfer to the intensive care unit (ICU) if necessary.

**Table 1:** Characteristics of included studies.

**Study identifier**	**Country**	**Population description**	**n PAP**	**Age (years)**	**Female %**	**Severity criteria**	**Non-mild (%)**
Abu-El-Haija (13)	USA	First PAP	165	DIAP: 13.7 (7.5–15.8); non–DIAP: 13.5 (10.0–15.9)	52.7	NASPGHAN	20.0
Antunes (14)	Portugal	PAP	37	NA	59.5	revised Atlanta	24.3
Berney (15)	Italy	PAP	24	10.8 (1–15)†	57.1	OF, ICU	20.8
Bierma et al. (16)	Australia, Netherlands	PAP	175	12.5 (9.2–15.6)	48.6	OF, ICU, local complications, need for pancreatic surgery, death	28.6
Birimberg-Schwartz (17)	Canada	First PAP	223	11 ± 4.8	50.2	NASPGHAN	16.1
Boskovic (18)	Serbia	First PAP	36	10.1 ± 4.7	58.3	revised Atlanta	44.4
Chang et al. (19)	Taiwan	First PAP	180	8.2 (0.2–17)	56.1	Atlanta	28.3
Coffey et al. (20) derivation cohort	Australia	PAP	73	11.6 (8.0–13.7)	37.0	OF, ICU, local complications, need for pancreatic surgery, death	34.2
Coffey et al. (20) validation cohort	Australia	PAP	58	15.1 (11.2–17.2)	60.3	OF, ICU, local complications, need for pancreatic surgery, death	24.1
DeBanto et al. (21) criterion group	USA	PAP ≤ 16 years	202	8.9 ± 1.1	NA	OF, local complications, need for pancreatic surgery, death	19.8
DeBanto et al. (21) validation group	USA	PAP ≤ 16 years	99	9.4 ± 1.5	NA	OF, local complications, need for pancreatic surgery, death	12.1
Fabre et al. (22)	France	First PAP	48	10.8 (2.1–19.5)†	47.9	Atlanta	27.1
Farrel et al. (23)	USA	First PAP	73	NA	64.4	NASPGHAN	30.1
Farrel et al. (23) derivation cohort	USA	First PAP	46	13.7 (9.1–16.2)	47.8	NASPGHAN	21.7
Farrel et al. (23) validation cohort	USA	First PAP	25	14.2 (11.1–17.3)	48.0	NASPGHAN	24.0
Fonseca Sepúlveda (24)	Colombia	PAP	130	11.4 ± 3.8	62.3	Atlanta	29.2
Galai et al. (25)	Israel	PAP ≥ 6 month follow–up	117	13.2 (7.0–15.9)	52.1	revised Atlanta	12.8
Guerrero-Lozano (26)	Colombia	PAP	30	NA	NA	revised Atlanta	NA
Hao (27)	China	PAP	159	6.2 ± 3.3	46.2	revised Atlanta	53.5
Hashimoto et al. (28)	Japan	PAP	37	6 (5–12)	59.5	OF, local complications, need for pancreatic surgery, death	56.8
Hornung (29)	USA	First PAP	176	NA	NA	NASPGHAN	22.2
Izquierdo et al. (30)	Colombia	PAP, CECT within 48h	30	10.5 ± 3.5	73.3	OF, local complications, need for pancreatic surgery, death	33.3
Izquierdo et al. (30)	Colombia	PAP	130	mild: 12 (7–17); M/SPAP: 11 (3–18)	62.3	OF, local complications, need for pancreatic surgery, death	29.2
Kandula (31)	USA	First PAP, ≤ 3 years	87	1.7 (0–2.9)†	48.3	OF, local complications, death	3.8
Kaur et al. (32)	India	PAP	134	11.9% <5; 34.3% 5–10; 40.3% 10–15; 13.4% 15–20	NA	NASPGHAN	42.5
APPLE (33–38)	mostly Hungary	PAP	45	11.7 (3–18)†	48.9	revised Atlanta	13.3
Lautz et al. (39)	USA	PAP	211	10.9 ± 4.9	47.9	OF, local complications, need for pancreatic surgery, death	26.5
Li (40)	China	First PAP, CECT on admission	107	9.3 (2.1–15.3)	45.8	revised Atlanta	25.2
Mehta (41)	USA	PAP	121	12.1 ± 4.6	60.3	NA	17.4
Nauka et al. (42)	USA	PAP	79	14 (9.5–16)	41.8	NASPGHAN	21.5
Orkin (43)	USA	First PAP ≤ 21 years	114	NA	NA	NA	NA
Parian (44)	Philippines	PAP	28	11.5 ± 4.1	NA	NA	NA
Pezzili et al. (45)	Italy	PAP	50	10.5 (2–17)†	50.0	Atlanta	18.0
Sag (46)	Turkey	First PAP	63	9.6 ± 4.8	50.8	NASPGHAN	46.0
Sánchez-Ramírez (47)	Mexico	PAP	55	10.5 ± 1.6	49.1	NA	NA
Suzuki et al. (48) criterion group	Japan	PAP (but 2–fold enzyme elevation)	145	7.3 (0.8–17)‡	60.7	OF, local complications, need for pancreatic surgery, death	6.9
Suzuki et al. (49) validation group	Japan	PAP (but 2–fold enzyme elevation)	131	7.7 ± 4.3	51.9	revised Atlanta	9.9
Szabo et al. (50) derivation group	USA	PAP ≤ 21 years	284	12.7 ± 4.9	50.0	ICU, local complications, respiratory complications (OF, oedema, pleural effusion), need for pancreatic surgery, death	19.0
Szabo et al. (50) validation group	USA	PAP ≤ 21 years	165	12.9 ± 5.2	58.2		NA
Thavamani et al. (51)	USA	PAP ≤ 21 years (CP excluded)	39,805	15.2 ± 4.7	59.2	revised Atlanta	4.0
Vitale et al. (52)	USA	First PAP ≤ 21 years	118	mild: 13.5 (10.2–15.9); M/SPAP: 13.8 (7.9–15.9)	47.5	NASPGHAN	18.6
Walker et al. (53)	UK	First PAP	59	13 (0.1–17)†	50.9	revised Atlanta	37.3
Wetherill (54)	UK	First PAP	37	14 (4–17)†	48.7	OF, local complications	35.1
Zheng et al. (55)	China	PAP	111	8.2 ± 3.3	53.2	NASPGHAN	13.5

Age is given as mean ± standard deviation, or median (interquartile range), unless otherwise indicated. †, median (range); ‡, mean (range). In the severity criteria column; most commonly “NASPGHAN” (2017 North American Society for Pediatric Gastroenterology; Hepatology; and Nutrition Pancreas Committee criteria); “Atlanta” (1992 Atlanta classification) and “revised Atlanta” (2012 revision of the Atlanta classification) are given; if not; the factors are provided based on which cases were classified as non-mild. CECT, contrast-enhanced computed tomography; CP, chronic pancreatitis; DIAP, drug-induced acute pancreatitis; h, hours; ICU, intensive care unit admission; M/SPAP, moderate or severe pediatric acute pancreatitis; n, total number; NA, not available; OF, organ failure; PAP, pediatric acute pancreatitis.

There are multiple proposed score systems that aim to predict which patients will develop M/SPAP. Those most widely examined are the modified Glasgow criteria (56), the Ranson criteria (57) and the Pediatric Acute Pancreatitis Severity (PAPS) score (21), all mainly based on laboratory parameters determined within the first 48 h. But many others are tested and proposed, involving variables such as blood urea nitrogen (BUN), white blood cell count (WBC), albumin, hemoglobin, among else (23, 50, 52). Still, there is no single pediatric-specific predictive value or score system that can be recommended (9). What is more, there are no comprehensive systematic reviews assessing the association between factors determinable on-admission and PAP severity.

Our aim was to perform a systematic review and meta-analysis of available predictive score systems and on-admission differences between severity groups in order to summarize the existing data and possibly shed light on the early identification of these patients.

## Materials and methods

### Protocol and reporting

The pre-study protocol was registered with PROSPERO, under the registration number: CRD42022307271. No deviations were made from the previously registered protocol. The findings are reported in this article according to the Preferred Reporting Items for Systematic Reviews and Meta-Analyses (PRISMA) Statement (58).

### Eligibility criteria

Studies were considered eligible regardless of study design (interventional and observational, retro- and prospective) in case they included at least 10 participants with PAP and presented data on any on-admission factors (any, e.g., demographic, symptom-related, laboratory values, imaging results, etc.) in different severity groups of the disease. Severity definitions used by the individual studies were accepted, but studies using different classifications were only pooled together if these classifications were comparable. In the end, almost all studies used severity classification systems based on the development on local complications and organ failure (sometimes supplemented with ICU admission), the two most common ones being the revised Atlanta classification (59) and the 2017 North American Society for Pediatric Gastroenterology, Hepatology, and Nutrition (NASPGHAN) criteria (60). These both form severity categories based on complications, according to the same principle: patients with organ failure lasting 48 h or more are categorized as severe; with transient (<48 h) organ failure, or local complications (acute peripancreatic fluid collection, pancreatic pseudocyst, acute necrotic collection or walled-off necrosis) or systemic complications (i.e., exacerbation of comorbidity), moderate; with none of the above, mild. Few older studies used the original Atlanta classification, which separates mild and severe pancreatitis, the latter mostly covering the moderate and severe disease courses in the newer classifications (61).

Our initial plan was to also compare severe and non-severe cases, but due to the low number of severe cases in the identified studies, data was rarely presented separately for these patients, thus only mild vs. M/SPAP comparison could be performed. Factors collected within 48 h of admission were accepted to be “on-admission.” Acute pancreatitis was defined as the presence of at least two of the following three criteria: abdominal pain; elevation in serum amylase or lipase reaching at least three-times the upper limit of the normal threshold; characteristic imaging findings.

The PECO (Population-Exposure-Control-Outcome) framework of our systematic review and eligible studies were:

P: PAP (≤18 years old).
E&C: any on-admission factor (demographic, laboratory, imaging, etc.).
O: PAP severity (mild, moderate, severe, non-mild, non-severe).

### Systematic search and selection

A systematic search was conducted on the 14^th^ February, 2020 in MEDLINE (*via* PubMed), EMBASE and CENTRAL with the following search key: “acute AND (pediatric OR pediatric OR children) AND pancreatitis AND (severe OR mild OR severity).” No restrictions were imposed on the search. Search results were imported into EndNote X9 (Clarivate Analytics, Philadelphia, PA) and selected according to a predefined set of criteria by two independent reviewers. In case of any disagreements, an independent third reviewer made the decision to include the study. The selection process was visualized using a PRISMA flow diagram.

### Data extraction

Data was extracted from eligible articles into a standardized Excel sheet and validated by an independent second reviewer (KO). The collected data included items related to study characteristics, the investigated study population, any exposures and controls investigated and outcomes (severity criteria used, number with mild, moderate and severe disease) as detailed in the pre-study protocol. In case of multiple reports of the same outcome in overlapping populations, the higher patient number was favored.

### Statistical analysis

For any on-admission factor examined in a comparable manner by at least three articles, we conducted a meta-analysis using a random-effects model. To estimate the between study heterogeneity we applied the Restricted maximum-likelihood estimation in case of continuous outcomes and the Mantel-Hanszel method with the Paule-Mandel estimator in case of dichotomous outcomes. We calculated pooled risk ratios (RR) for dichotomous and mean differences (MD) for continuous variables with 95% confidence intervals (CI), and visualized the results using forest plots. To quantify existing statistical heterogeneity, we performed Chi^2^ tests (using a *p* < 0.1 to indicate statistically significant heterogeneity), and calculated I^2^ values (0 to 40%: might not be important; 30 to 60%: may represent moderate heterogeneity; 50 to 90%: may represent substantial heterogeneity; 75 to 100% considerable heterogeneity). It should be pointed out that in the case of several laboratory variables (serum lipase, amylase, C-reactive protein (CRP), BUN and creatinine) more than half of the data had to be converted from medians with interquartile ranges to means with standard deviations using the default setting of the metacont function (62), in order to perform meta-analytical calculations.

In case predictive variables or score systems were reported in a way that the number of true and false negatives and positives were ascertainable in a sufficient number of studies, a random effects meta-analysis was performed and a hierarchical summary receiver operating characteristic (HSROC) curve was computed with a 95% confidence region and a 95% prediction region, using a bivariate model (63). Although this method is currently deemed most valid in case of a low number of studies, its results might be more limited below 10 studies, which was not achieved in our paper (64). In case of at least 10 studies for a given analysis, we created and visually assessed funnel plots and performed Egger's test to assess the possibility of publication bias.

All calculations were be performed using R: A language and environment for statistical computing [R version 4.1.2, “mada” and “meta” packages, R Core Team (65), Vienna, Austria].

### Risk of bias

To assess the risk of bias in the included studies, we used the Quality in Prognostic Studies (QUIPS) tool, as recommended by the Cochrane Collaboration (66). Two independent reviewers conducted the assessment (MFJ and KO).

## Results

### Study selection

**Figure 1:**
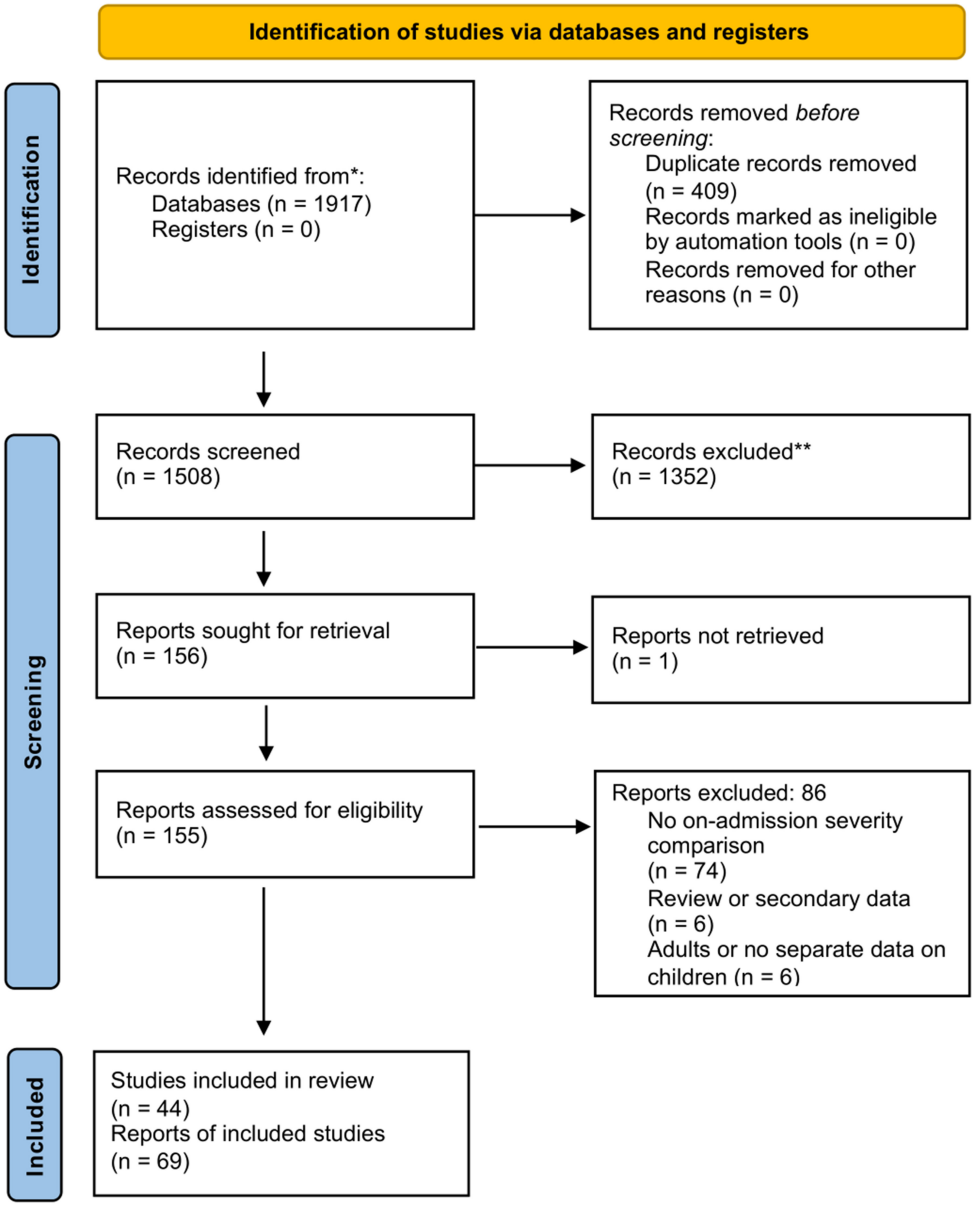
PRISMA flow diagram. The selection of reports is visualized. *n*: number.

The systematic search retrieved 1,917 records, of which 44 studies, reported on by 69 records were found eligible for inclusion. The selection process is visualized on Figure 1.

### Characteristics of included studies

Among the 44 included studies, most were retrospective cohort studies, with the exception of three prospective studies (13, 52, 67) and two that were in part prospective (23, 29). Studies either examined all PAP patients, or excluded recurrent episodes. As explained in the ‘Methods' section of this manuscript, almost all studies used the 2017 NASPGHAN or the revised Atlanta classification or a comparable method for forming severity categories. In the majority of studies, 70–80% of cases were classified as mild. Study report citations can be found in our Supplementary Table 1.

### Synthesis of results

#### Primary outcome

##### Predictive score systems, predictive parameters

**Figure 2:**
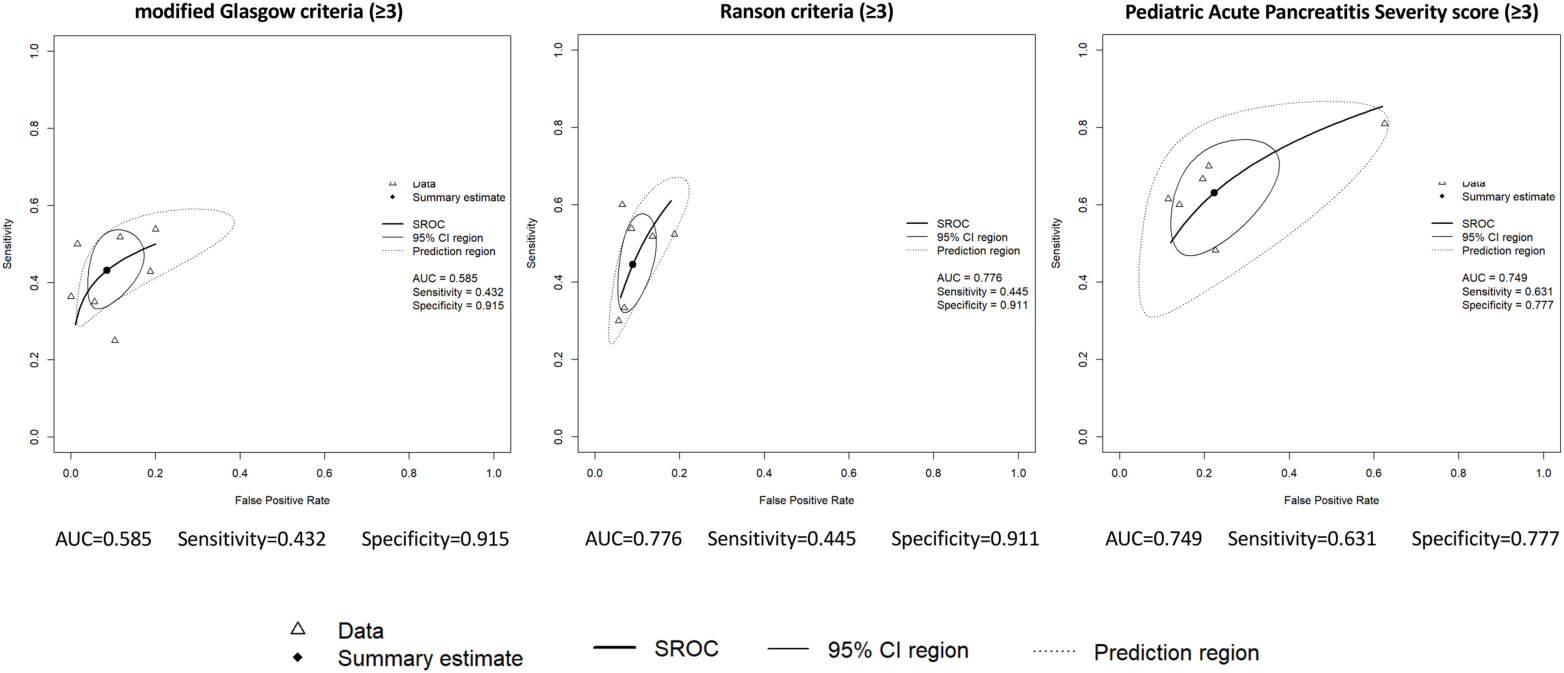
Hierarchical summary receiver operating characteristic (HSROC) curves. Data are presented for the following predictive score systems: modified Glasgow criteria, Ranson criteria, Pediatric Acute Pancreatitis Severity score. The values used as cut-off for indicating a moderate or severe disease are given in brackets. Triangles represent data obtained from individual studies, rectangles represent the summary estimates, bold solid line the summary receiver operating curve, solid line the 95% CI region, dotted line the prediction region. AUC, area under the curve; CI, confidence interval; SROC, summary receiver operating characteristic curve.

We were able to perform meta-analytical calculations for the three most widely examined predictive score systems: the modified Glasgow criteria, the Ranson criteria and the PAPS score. The produced HSROC curves, AUC values of these systems, their sensitivity and specificity for predicting M/SPAP with a score of 3 or higher are presented on Figure 2, additional summary estimates can be found in the Supplementary Figures 1–6. AUC values could also be pooled from three studies for the modified Glasgow score: 0.76 (95% CI: 0.61–0.92) (Supplementary Figure 7).

Other prognostic scores and parameters for which information was available on predictive performance measures, but not enough data was provided to conduct meta-analytical calculations are narratively summarized in Table 2.

**Table 2:** Summary of predictive performance parameters presented by the included studies.

**Predictive score/factor**	**Studies (ref)**	**Assessed within:**	**AUC**	**Cut-off**	**Sens (%)**	**Spec (%)**	**PPV (%)**	**NPV (%)**
Computed Tomography Severity Index (CTSI)	3 (27–29)	48 h	0.64–0.90	score ≥ 4	50–81	78–86	61–71	**98–99**
Pediatric JPN score	2 (31, 32)	48 h		score ≥ 3	80–83	**96–98**	62–77	50–90
Lipase + WBC + albumin	1 (17)	24 h	0.76–0.77	best performance	68	71		
Hemoglobin <13 g/dL and/or BUN ≥12.5 mg/dL	1 (33)	24 h		1 or both present	81.5	64.1		**89.3**
Lipase >7xULN and Ca trough ≤ 2.15 mmol/L	1 (36)	48 h		both present	46	**89**	65	79
Lipase	4	24 h	0.61–0.80	≥ 7xULN	**82–94**	23–56		
	(17, 34–36)							
	1 (36)	48 h		50% decrease on day 2	73	54	46	79
				≥ 7xULN + 50% decrease day 2	67	79		
Amylase	1 (34)	24 h	0.70	≥3xULN	62.5	80.0		
Hemoglobin	1 (34)	24 h	0.70	≥ 143 g/L	**85.7**	43.5		
WBC	2 (17, 37)	24 h	0.59–0.63					
	1 (38)	48 h	0.79	>17 G/L	68.2	81.1	68.2	81.1
CRP	2	24 h	0.73–0.39	>27.5 mg/L	68.2	81.1		
	(37, 39)							
	1 (38)	48 h	**0.92**	>108 mg/L	**91.0**	83.8		
Albumin	2 (17, 37)	24 h	0.71–0.80					
	2	48 h	**0.85**	<34 g/L	**91.0**	75.7	69.0	**93.3**
	(38, 40)							
				<28 g/L	41.0	**90.4**	80.0	62.2
BUN	2	24 h	0.73–0.75	≥13 mg/dL	63–68	73–81	52–72	**84–91**
	(16, 18)							
	1 (40)	48 h		≥20 mg/dL	48.8	**85.0**	63.6	75.5
Calcium	2 (36, 40)	48 h		<2.1 mmol/L	47.6	81.1	60.6	71.7
				trough ≤ 2.15 mmol/L	59	81	60	80
Dyspnoea	1 (40)	48 h		present	23.5	**98.4**	**85.7**	76.5
Pleural effusion	1 (40)	48 h		present	49.0	**95.3**	80.6	82.5

The left column indicates the factor, combination of factors or group of factors examined. The second column presents the number of enrolled studies presenting data on said factor, with their references. When data is not presented as a single value, but instead with a dash, that indicates the range of values observed by the available studies. Predictive values indicating good performance (using an arbitrary threshold of 0.85) appear in bold to ease overview. AUC, area under the curve; BUN, blood urea nitrogen; CRP, C-reactive protein; NPV, negative predictive value; PPV, positive predictive value; Sens, sensitivity; Spec, specificity; ULN, upper limit of normal; WBC, white blood cell count.

#### Secondary outcomes

##### Demographic factors, previous pancreatitis

We were able to perform quantitative syntheses for differences in age and previous PAP. We found no statistically significant difference in the age of onset between patients with mild and M/SPAP. There was a tendency of younger onset in the M/SPAP group, MD: 1.08 years younger (95% CI: 2.21 years younger to 0.05 years older; I^2^ = 72.5%, *p* < 0.001; Supplementary Figure 8). While also no statistically significant difference was found between genders, there was a tendency of less M/SAP cases among females (RR: 0.87, 95% CI: 0.73–1.03; I^2^ = 0%, *p* = 0.808; Supplementary Figure 9).

We found a history of previous PAP to be associated with an increased rate of M/SPAP (RR: 1.64, 95% CI: 1.21–2.23; I^2^ = 0%, *p* = 0.446; Supplementary Figure 10).

Multiple studies reported on weight differences between mild and M/SPAP, although in altering ways, rendering meta-analytical calculations unfeasible. Generally speaking, most studies noted no significant differences between groups. Of note, Thavamani 2021 analyzed an inpatient database in the United States covering a high number of patients and found both undernutrition and obesity to be associated with increasing PAP severity (51).

##### Etiology

**Figure 3:**
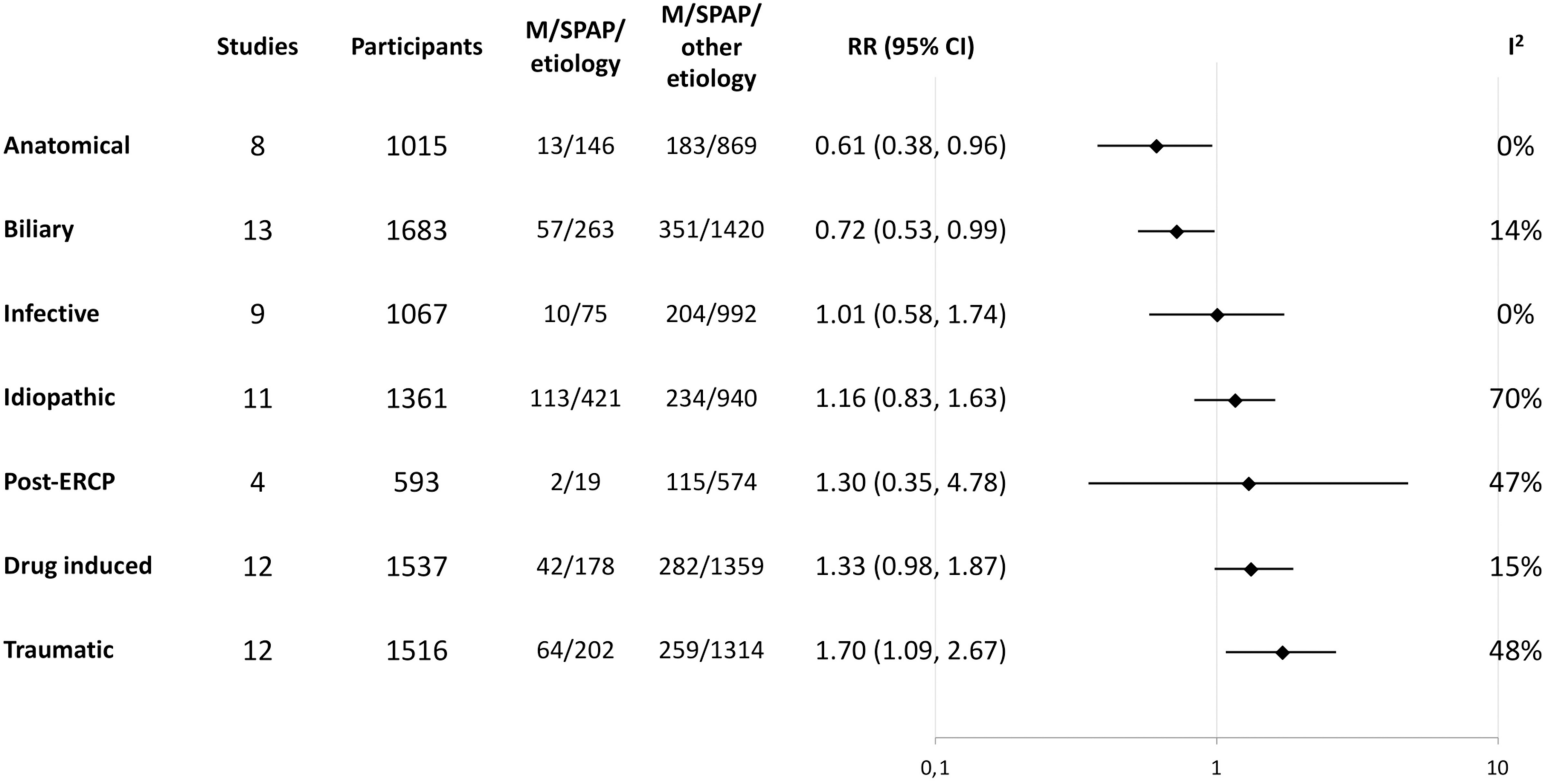
Summary of moderate or severe disease course risk with different etiologies. Random-effect meta-analysis results are summarized for the etiological factors and ordered according to effect size. The “Studies” and “Participants” columns indicate the total number of studies and participants in the meta-analysis, while the next two columns indicate the number of events (moderate or severe course) in those with the analyzed etiology and those without. CI, confidence interval; M/SPAP, moderate or severe pediatric acute pancreatitis; RR, risk ratio.

We were able to perform quantitative syntheses assessing the risk of M/SPAP for the following etiologies or risk factors: abdominal trauma, anatomical malformations, associated drugs, biliary obstruction, idiopathic PAP, infective PAP, PAP following endoscopic retrograde cholangiopancreatography (ERCP). PAP related to anatomical malformation or biliary obstruction was associated with a lower likelihood of M/SPAP, while the proportion of M/SPAP was higher next to drug-induced or traumatic etiologies. Effects are summarized on Figure 3. The individual forest plots for these comparisons can be found in the Supplementary Figures 11–17.

Metabolic, systemic and genetic etiologies were also reported on by multiple studies, but their definitions were either substantially different or unascertainable, so meta-analyses were not performed for these etiological factors. Even though patient numbers were usually low for these etiologies in most studies, there was a tendency of more M/SPAP cases in patients with underlying PAP-associated systemic diseases, and no such tendency was seen with metabolic causes and genetic or familial cases (16, 19–21, 48).

##### Differences in laboratory parameters

We were able to perform quantitative syntheses for the following on-admission serum laboratory parameters: lipase, amylase, WBC count, lactate dehydrogenase (LDH), CRP, glucose, BUN, albumin, aspartate transaminase (AST) and creatinine (Supplementary Figures 18–27).

M/SPAP was associated with significantly higher on-admission WBC (+4.86 G/L, 95% CI: +3.14 – +5.17 G/L; I^2^ = 58.9%, *p* = 0.013), LDH (+876.8 U/L, 95% CI: +25.4 – +1,728.1 U/L; I^2^ = 73.0%, *p* = 0.011), CRP (+15.2 mg/L, 95% CI: +9.1 – +21.3 mg/L; I^2^ = 10.7%, *p* = 0.347), glucose (+0.73 mmol/L, 95% CI: +0.28 – +1.17 mmol/L; I^2^ = 0.0%, *p* = 0.617) and BUN (+0.96 mmol/L, 95% CI: +0.02 – +1.91 mmol/L; I^2^ = 78.5%, *p* = 0.003). There was a tendency of higher lipase (+426.4 U/L, 95% CI: −244.3 – +1097.1 U/L; I^2^ = 74.3%, *p* < 0.001) and amylase (+125.2 U/L, 95% CI: −76.1 – +326.4 U/L; I^2^ = 62.2%, *p* = 0.021) values and lower albumin (−3.34 g/L, 95% CI: −8.20 – +1.52 g/L; I^2^ = 81.1%, *p* = 0.013) on admission. No difference was found between mild and M/SPAP in on-admission AST (−11.3 U/L, 95% CI: −194.8 – +172.2 U/L; I^2^ = 97.3%, *p* < 0.001) and creatinine (+0.48 μmol/L, 95% CI: −4.35 – +5.30 μmol/L; I^2^ = 0.0%, *p* = 0.528).

The narrative review of other laboratory parameters not eligible for quantitative synthesis was beyond the scope of our paper.

##### Additional outcomes, prediction of severe cases

Izquierdo 2018 and Lautz 2011 performed retrospective reviews of patients with PAP who had CT investigations on admission (within 48 and 72 h, respectively). While Lautz 2011 found the presence of necrosis to be significantly associated with PAP severity (42.3% vs. 10.5% M/SPAP in those with and without necrosis, *p* = 0.002), in the work by Izquierdo et al. only parenchymal necrosis >30% showed such an association (39, 68). Galai et al. and Pezzili et al. reported on symptom duration, which was not significantly different between groups in their cohorts (25, 45). In Nauka 2019, systemic inflammatory response syndrome (SIRS) on admission was significantly associated with M/SPAP (odds ratio: 3.23, 95% CI: 1.01–9.78, *p* = 0.038) (42).

Only three studies presented detailed data on admission differences between patients with severe and non-severe PAP. In Hao 2018, previous PAP was significantly associated with severe disease course. Mehta and colleagues found an opposite tendency: none of their five severe cases had previous episodes vs. 51 and 63% of mild and moderate cases. In Li 2018, patients with severe PAP had significantly higher WBC, neutrophil count and CRP on admission, as well as a significantly higher CTSI score.

### Risk of bias

Risk of bias assessment results, separated according to above results subsections, are available in the Supplementary Figures 28–30.

## Discussion

In this systematic review and meta-analysis we assessed the association between on admission factors and the severity of acute pancreatitis in the pediatric age group. Due to the nature of the available studies we were able to compare the on-admission presentation of mild and M/SPAP. Although the definitions of M/SPAP minimally varied in the included studies, it generally represents patients who developed local or systemic complications or organ failure.

Our main finding and the foremost merit of our paper is the meta-analysis of the most commonly used severity prediction score systems and the rigorous narrative review and summary of other examined and proposed variables and scores for PAP severity prediction.

The most widely explored severity prediction scores were the modified Glasgow criteria, the Ranson criteria and the PAPS score. The modified Glasgow and Ranson criteria, initially developed for adult-onset acute pancreatitis demonstrated good specificity for predicting M/SPAP (both around 91–93%), but subpar sensitivity (43 and 45%, respectively). In 2002, DeBanto and colleagues developed the pediatric specific PAPS score largely based on these two criteria, also arguing that sensitivity and NPV are more important in this case, so that no severe cases are missed (21). They were able to achieve an improvement in sensitivity in their cohort (67–70%), with lower specificity (79–81%). But overall, the meta-analysis of six studies examining the PAPS score in a comparable manner found more modest predictive metrics: a sensitivity of 63% and a specificity of 78%. To conclude, none of the above scoring systems have an acceptable sensitivity for predicting M/SPAP. As DeBanto and colleagues phrased it, this would be crucial, since the rationale is that all or almost all patients with M/SPAP should be identified so we can know when to be more vigilant.

But all is not lost – there are several other parameters that were examined (some of them even in multicentric settings and in multiple studies) that were reported to have acceptable or good sensitivity for predicting M/SPAP. Suzuki et al. modified the adult JPN scoring system to fit the pediatric age group and supplemented it with the age and weight thresholds used in PAPS score (69). In their criterion and validation groups they achieved good sensitivity (80–83%) and exceptional specificity (96–98%). Although very promising, as no other studies (be it dependent or independent) have further examined this score, the results should be handled with care. Multiple studies examined the severity predictive ability of lipase, a parameter not included in any of the mentioned score systems: its elevation above seven-times the ULN within 24 h showed a sensitivity of 82–94% in three separate studies. Hemoglobin is also not included in any of the above scores and it was not significantly different between groups in many of the cohorts. A possible reason behind this is that both its elevation and decrease are observed to predict M/SPAP – as Coffey et al. hypothesized, due to hypovolemia and due to hemorrhage –, with good sensitivity, albeit in few studies (20). Walker and colleagues also demonstrated good sensitivity for albumin <34 g/L and CRP > 108 mg/L within 24 h, although more modest CRP elevation showed poor sensitivity in another study. These parameters could either serve as an adjunct to the predictive scores with good specificity, or be used to develop new score systems with the goal of utilizing a single one that is optimal in all its predictive metrics.

There is also something to be said for the simplification of these scores. The modified Glasgow, Ranson, PAPS and JPN scores all rely on numerous parameters (all >8) some of which are expensive or difficult to assess (e.g., fluid sequestration, collecting arterial blood sample for partial oxygen tension) or unnecessarily invasive to children. And it should be pointed out that – although later extensively validated – the original Ranson criteria was developed on a modest 100 patients, involving all 11 objective parameters that correlated with serious illness or death (70). This later served as a basis for the Glasgow score, adapted with minimal modifications to the pediatric population in the form of the PAPS score. So there is no saying, that a less complicated set of parameters could not replace these existing combinations. Another important drawback, is that all four scores include parameters taken 48 h after admission, when on admission or within 24 h would clearly be preferable. While there are numerous promising simpler score system alternatives, as highlighted in the appropriate part of our “Results” section, these are rarely validated by other (especially independent) studies (although there are exceptions of course), and they usually fall short of the modified Glasgow and Ranson in terms of specificity and lipase > seven-times the ULN in terms of sensitivity.

Another key detail is that the existing and proposed scores are almost entirely based on laboratory parameters. The only non-laboratory parameter in the Ranson score and its derivates is age, which was transposed to be <7 years or <23 kilograms in the PAPS and pediatric JPN scores. DeBanto and colleagues introduced these cut-offs to define a lower limit of physiological reserves. In our meta-analysis, we found no significant age difference between mild and M/SPAP, which indicates that a simple threshold cannot be used, either because there is no age difference, or because multiple severity peaks exist. As Thavamani and colleagues found in their large-scale analysis, both undernutrition and obesity are associated with increased PAP severity (51).

There are a handful of studies that look to imaging results in the prediction of M/SPAP. The CTSI is based on the characterization of the inflammation and necrosis *via* contrast enhanced computed tomography (CECT). This method is established among adults, predicting severe pancreatitis with an around 86% sensitivity and 71% specificity, when performed within 48–72 h of admission (71). While some authors argue that performing a CECT should be a part of the routine evaluation of patients with pancreatitis, guidelines still do not recommend it, due to fiscal reasons, radiation and the existence of useful predictive scores (72–74). Availability and especially radiation are even greater concerns in a pediatric setting, thus the routine use of the CTSI is unlikely. Still, retrospective studies evaluated its performance in PAP, finding an around 80% specificity and conflicting results for sensitivity (22, 28, 39). It should be pointed out, that a retrospective approach is even more limited in this case, since the proportion of patients with PAP and available CECT results is low. An alternative could be ultrasound based severity prediction, which, although not routinely used, had some promising results in adults (75), but is yet to be examined in children.

Aside from laboratory and imaging results, not much else is taken into account in the available literature. Etiological factors for example – in our meta-analysis, traumatic and drug-induced etiologies were associated with a higher rate of M/SPAP, while a higher rate of mild cases was seen in children with anatomical malformations or PAP of a biliary origin. We also found a history of previous PAP to be associated with M/SPAP. These parameters could serve as promising additions to future score systems.

### Strengths and limitations

Perhaps the biggest strength of our work is that we do not know of any previous systematic review and meta-analysis in the topic. Another major strength is that we did not restrict our eligibility in terms of the factors assessed on admission – any that were detailed in the identified publications were reviewed, including demographic, etiological, laboratory, imaging symptom-related, etc. We also performed a meta-analysis of available predictive score systems that were examined in different publications with varying diagnostic metrics, thus we are able to give an estimation of their true predictive capabilities.

Among limitations, it should be stated that almost all studies were retrospective and this can influence some of our results: e.g., the performance of predictive systems or the availability of laboratory measurements might differ in a prospective setting. Since only three full-text articles were stated to be prospective, that didn't allow for the performance of subgroup analysis of only prospective studies. Another limitation is that – most likely due to the low number of severe cases – no severe vs. non-severe comparisons could be made. As disclosed in the “Statistical analysis” section of the manuscript, continuous data frequently had to be converted to means, which is limited in case of most laboratory variables, since these do not follow a normal distribution in PAP. Low patient numbers, such as in the case of infective and post-ERCP etiologies should also be noted, since it can reduce our confidence in these findings.

### Conclusions

None of the available scoring systems provide acceptable sensitivity and specificity for predicting which patients with pediatric pancreatitis will develop a moderate or severe disease course. The Ranson and modified Glasgow scores have the best specificity, but their sensitivity is subpar. Parameters such as lipase exceeding seven times the ULN could be used as an adjunct or added to future score systems to improve sensitivity, which is crucial in this case. Future scores should also strive for simplification and using only factors assessed on-admission or within 24 h. Non-laboratory parameters are rarely investigated, conversely, our analysis suggests that factors such as etiology and previous pancreatitis show an association with PAP severity. Major limitations of the current state of predictive score development are the retrospective study design, modest patient numbers and frequent non-validation of proposed scores by fellow researchers, which can only be improved by multi-center collaborative studies.

### Implications

**...for practice**: The Ranson and modified Glasgow scores provide the best specificity and lipase values > seven-times the ULN the best sensitivity for predicting which patients with PAP will develop complications. These patients should be monitored closely in order for prompt treatment initiation.

**...for research**: Our systematic review can serve as a basis for future predictive score system development. We highlight the importance of simplicity, using on-admission parameters and reaching this goal via forming international collaborations and investigating prospectively.

## Author contributions

Authorship was based on the criteria proposed by International Committee of Medical Journal Editors (ICMJE). MJ, AP, and AN drafted the conception of the work. MJ and KO conducted the data acquisition. ZS the data analysis. MJ and AP wrote the manuscript. All authors contributed to the interpretation of the data. All authors revised the manuscript critically for important intellectual content, approved the final version of the manuscript, and agree to be accountable for all aspects of the work.

## Funding

This study was supported by National Research, Development and Innovation Office Grant (FK138929) and the Cystic Fibrosis Trust Strategic Research Center Grant (NU000600), both awarded to AP.

## Conflict of interest

The authors declare that the research was conducted in the absence of any commercial or financial relationships that could be construed as a potential conflict of interest.

## Publisher's note

All claims expressed in this article are solely those of the authors and do not necessarily represent those of their affiliated organizations, or those of the publisher, the editors and the reviewers. Any product that may be evaluated in this article, or claim that may be made by its manufacturer, is not guaranteed or endorsed by the publisher.
