# Supplementary material for: Admission risk factors and predictors of moderate or severe pediatric acute pancreatitis: A systematic review and meta-analysis
Source: Front Pediatr. 2022 Sep 30;10:947545. doi: 10.3389/fped.2022.947545 (PMC9561825; doi:10.3389/fped.2022.947545)
Supplement: Supplementary file 1 [file Data_Sheet_1.pdf]

Supplementary Material

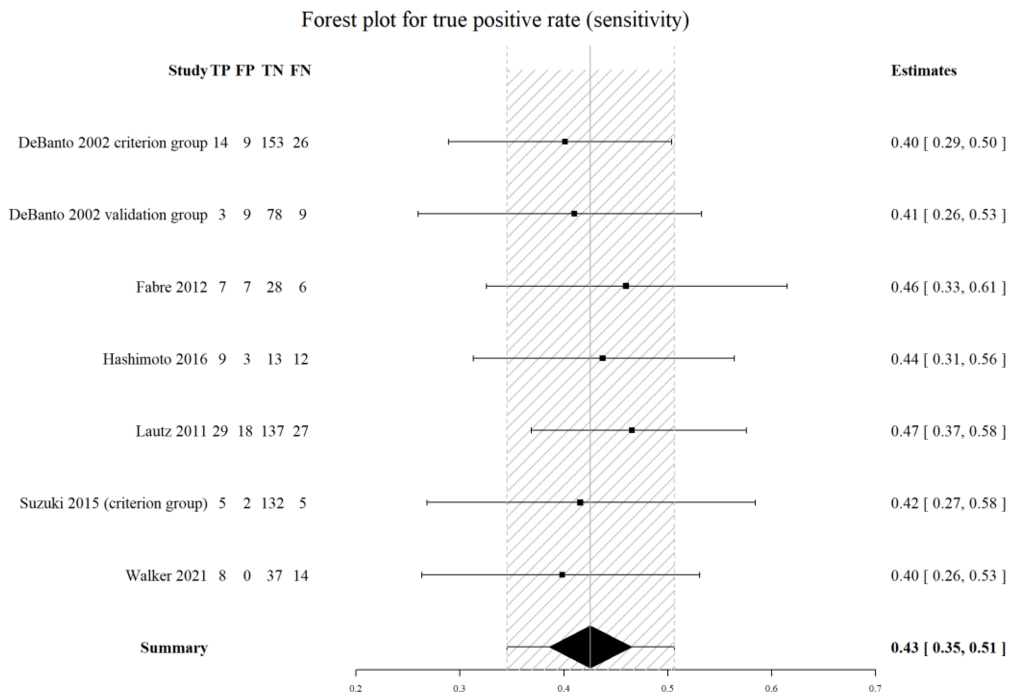

Supplementary Figure 1 Forest plot for sensitivity for modified Glasgow score predicting moderate or severe pediatric acute pancreatitis with a score of 3 or higher (TP: true positive; FP: false positive; TN: true negative; FN: false negative)

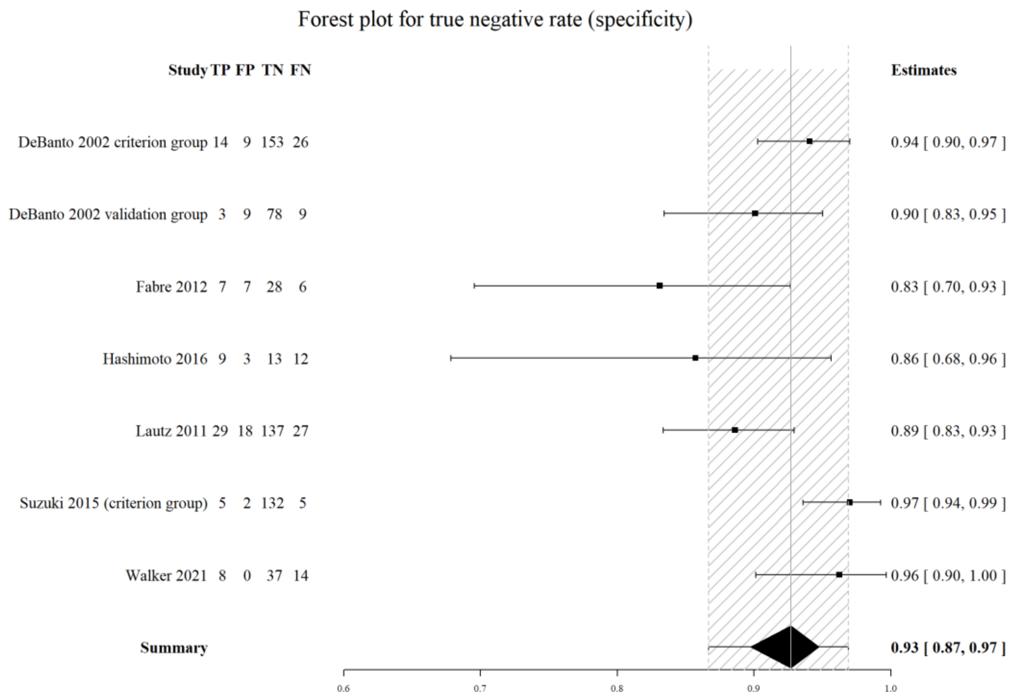

Supplementary Figure 2 Forest plot for specificity for modified Glasgow score predicting moderate or severe pediatric acute pancreatitis with a score of 3 or higher (TP: true positive; FP: false positive; TN: true negative; FN: false negative)

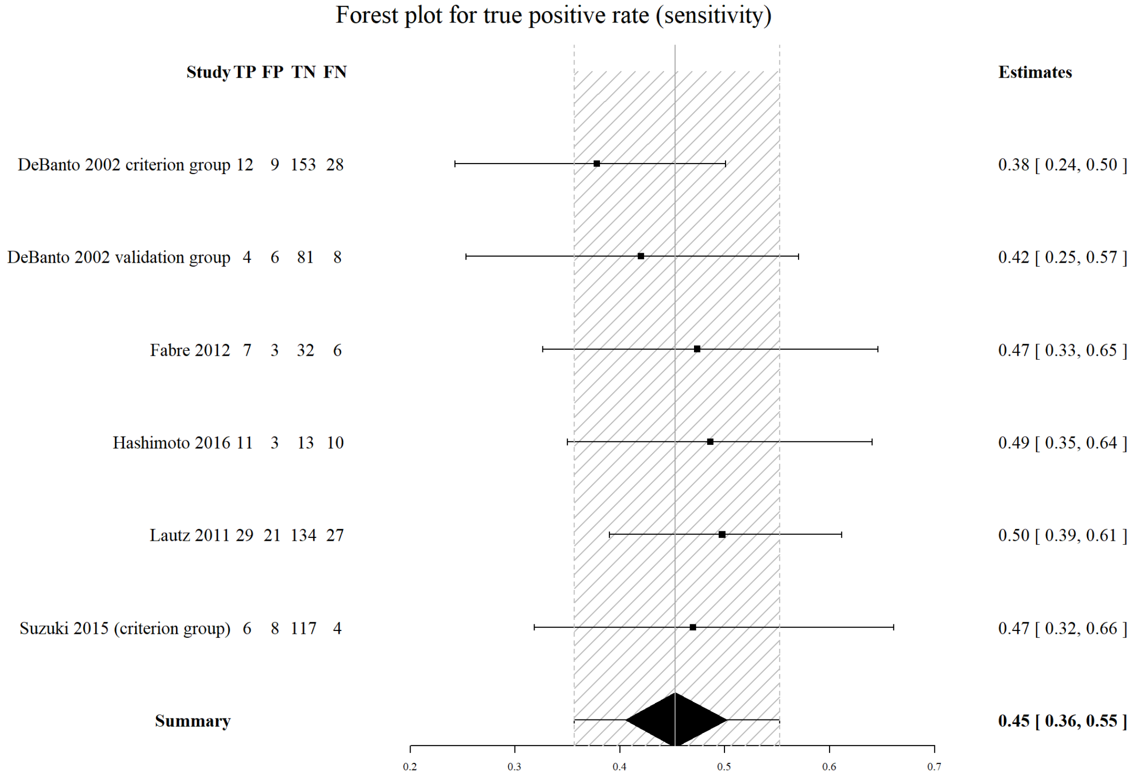

Supplementary Figure 3 Forest plot for sensitivity for Ranson criteria predicting moderate or severe pediatric acute pancreatitis with a score of 3 or higher (TP: true positive; FP: false positive; TN: true negative; FN: false negative)

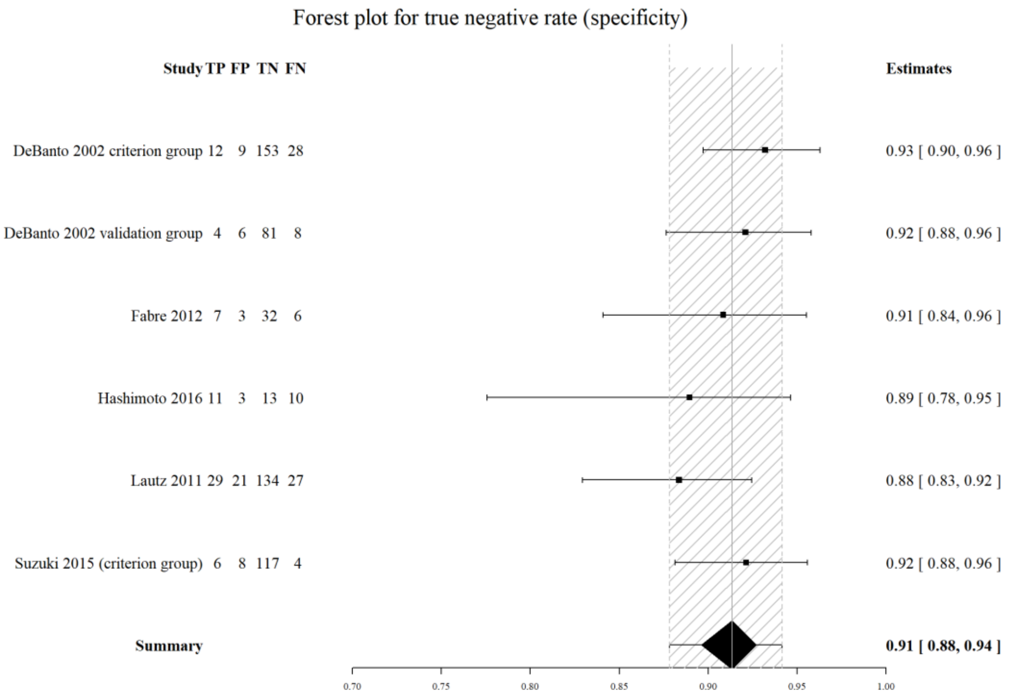

Supplementary Figure 4 Forest plot for specificity for Ranson criteria predicting moderate or severe pediatric acute pancreatitis with a score of 3 or higher (TP: true positive; FP: false positive; TN: true negative; FN: false negative)

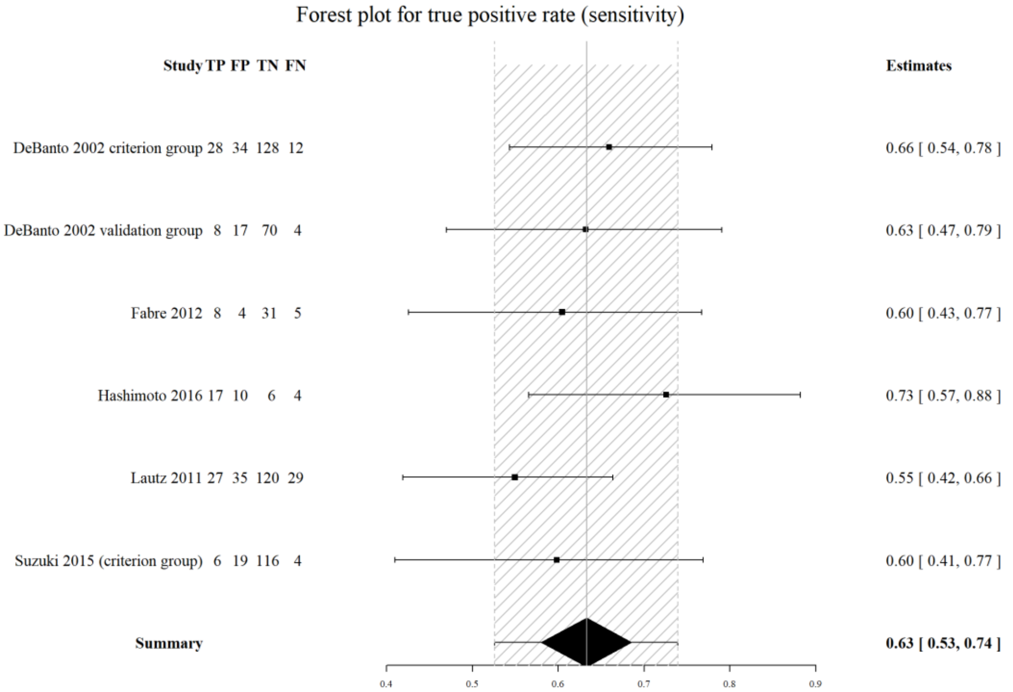

Supplementary Figure 5 Forest plot for sensitivity for Pediatric Acute Pancreatitis Severity (PAPS) score predicting moderate or severe pediatric acute pancreatitis with a score of 3 or higher (TP: true positive; FP: false positive; TN: true negative; FN: false negative)

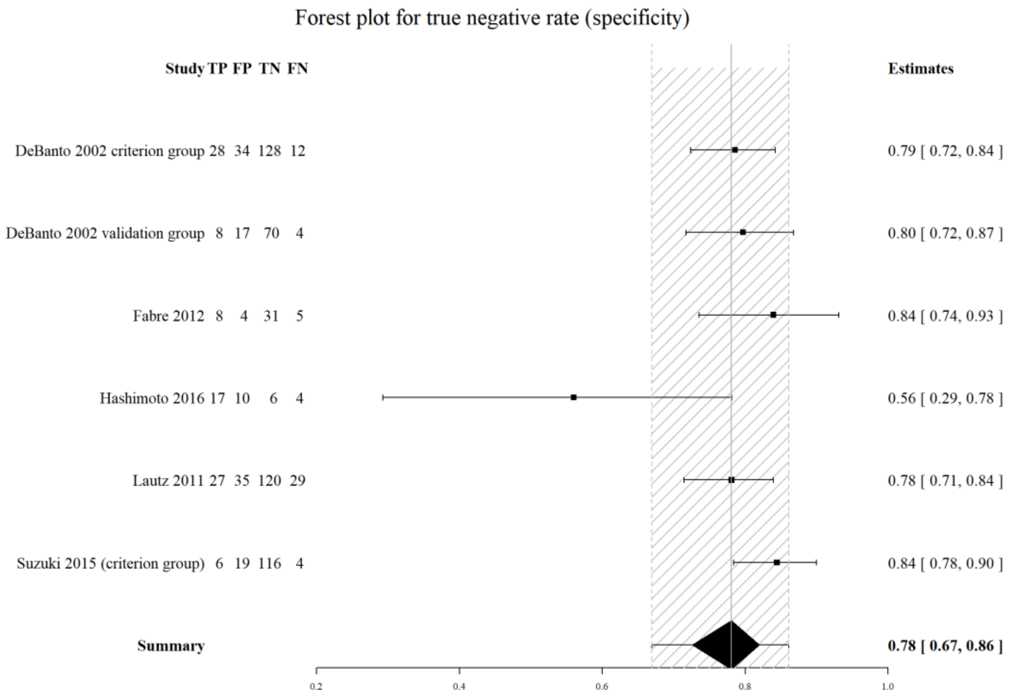

Supplementary Figure 6 Forest plot for specificity for Pediatric Acute Pancreatitis Severity (PAPS) score predicting moderate or severe pediatric acute pancreatitis with a score of 3 or higher (TP: true positive; FP: false positive; TN: true negative; FN: false negative)

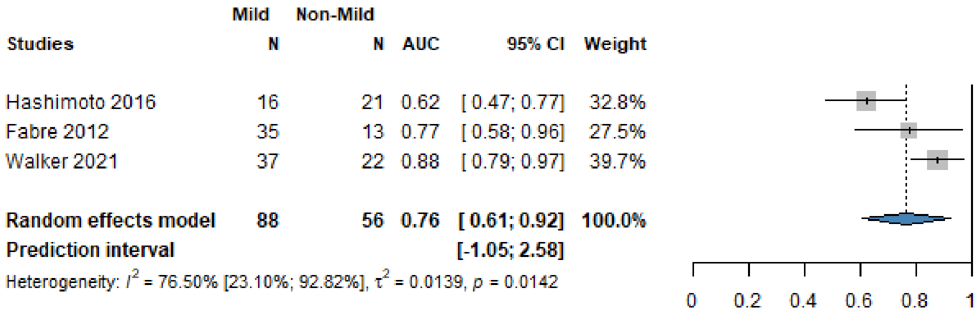

Supplementary Figure 7 Meta-analysis of AUC values for modified Glasgow score predicting moderate or severe pediatric acute pancreatitis with a score of 3 or higher (M/SPAP: moderate or severe pediatric acute pancreatitis; N: patient number; AUC: area under the curve; CI: confidence interval)

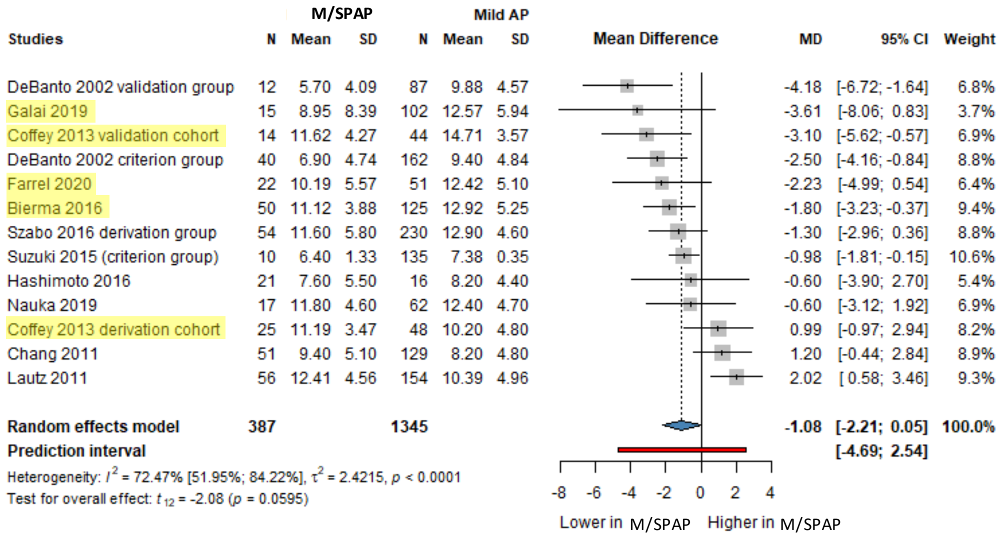

Supplementary Figure 8 Forest plot for age of onset of pediatric acute pancreatitis. A yellow background indicates that data had to be converted from medians with interquartile ranges to means with SD (AP: acute pancreatitis; M/SPAP: moderate or severe pediatric acute pancreatitis; N: patient number; SD: standard deviation; MD: mean difference; CI: confidence interval)

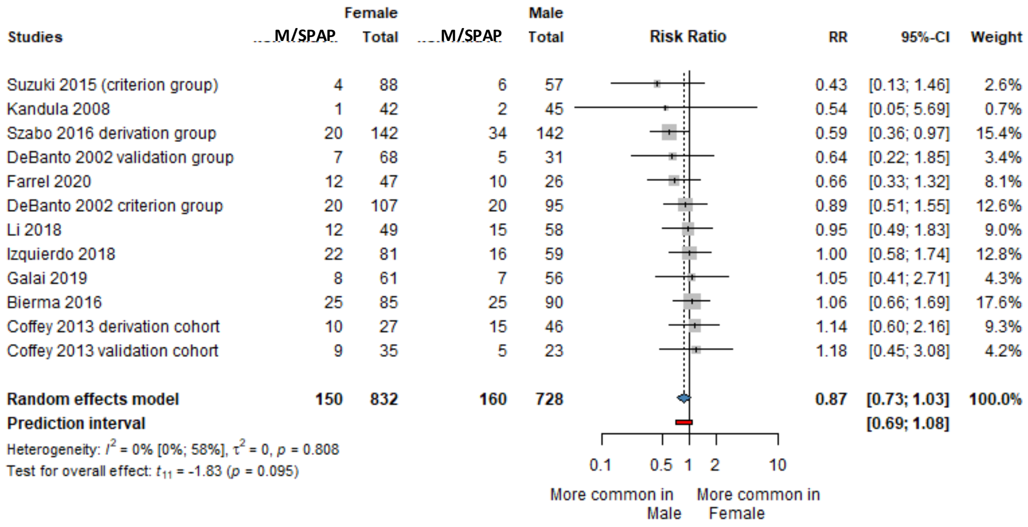

Supplementary Figure 9 Forest plot for gender in pediatric acute pancreatitis (M/SPAP: moderate or severe pediatric acute pancreatitis; RR: risk ratio; CI: confidence interval)

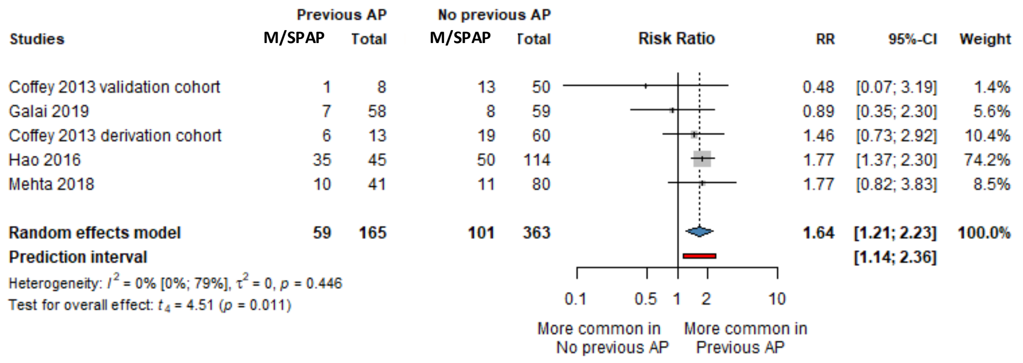

Supplementary Figure 10 Forest plot for previous acute pancreatitis in pediatric acute pancreatitis (AP: acute pancreatitis; M/SPAP: moderate or severe pediatric acute pancreatitis; RR: risk ratio; CI: confidence interval)

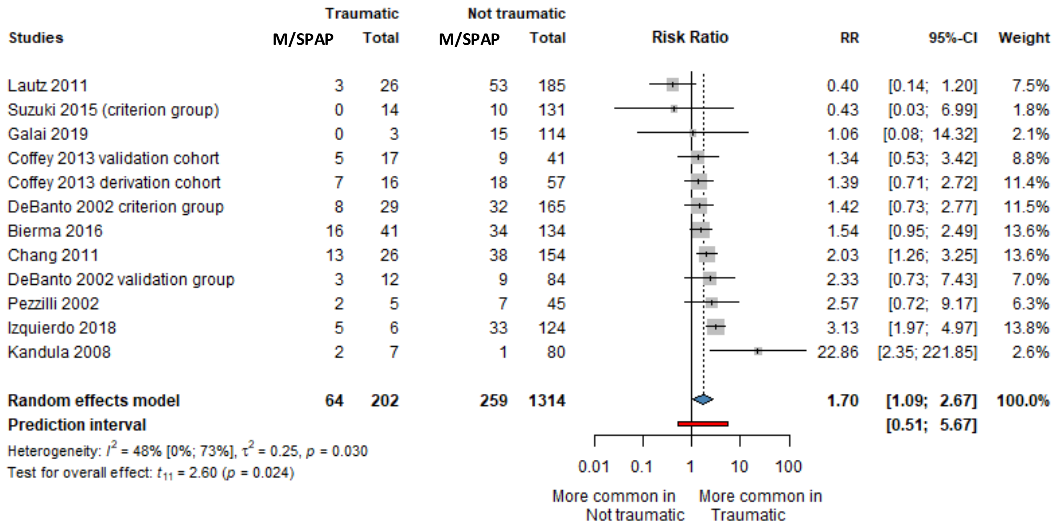

Supplementary Figure 11 Forest plot for abdominal trauma in pediatric acute pancreatitis (AP: acute pancreatitis; M/SPAP: moderate or severe pediatric acute pancreatitis; RR: risk ratio; CI: confidence interval)

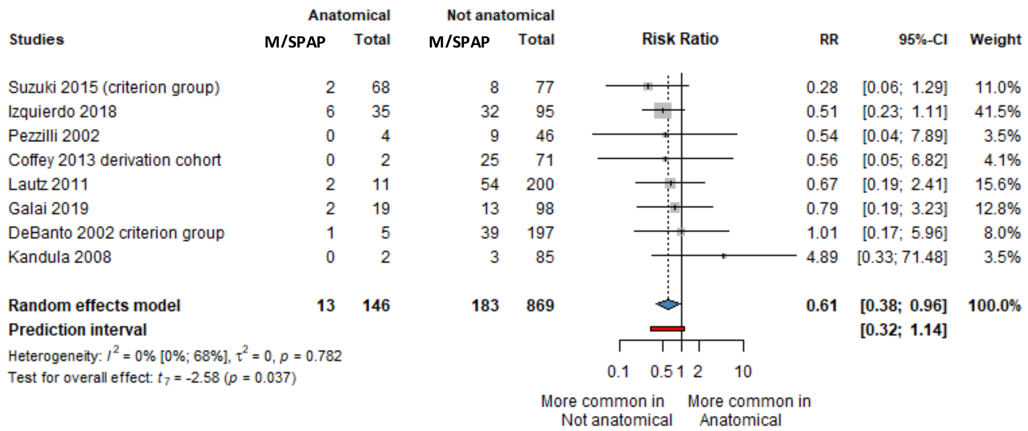

Supplementary Figure 12 Forest plot for anatomical malformations in pediatric acute pancreatitis (M/SPAP: moderate or severe pediatric acute pancreatitis; RR: risk ratio; CI: confidence interval)

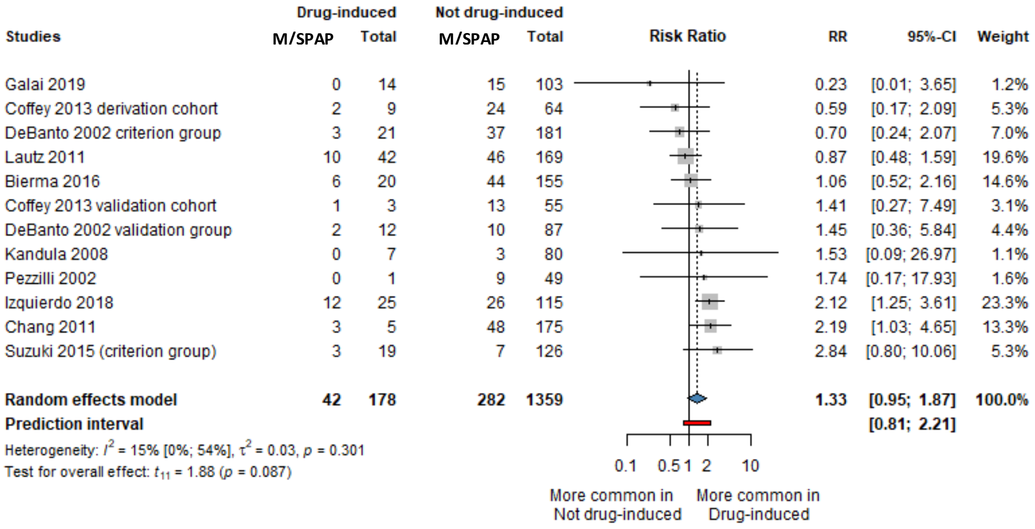

Supplementary Figure 13 Forest plot for drug-induced pediatric acute pancreatitis (M/SPAP: moderate or severe pediatric acute pancreatitis; RR: risk ratio; CI: confidence interval)

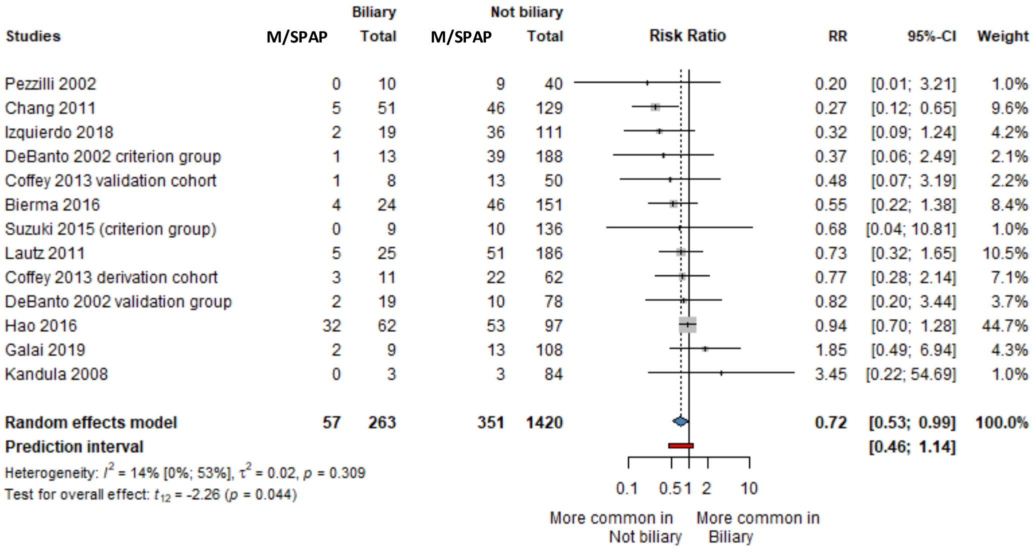

Supplementary Figure 14 Forest plot for biliary pediatric acute pancreatitis (M/SPAP: moderate or severe pediatric acute pancreatitis; RR: risk ratio; CI: confidence interval)

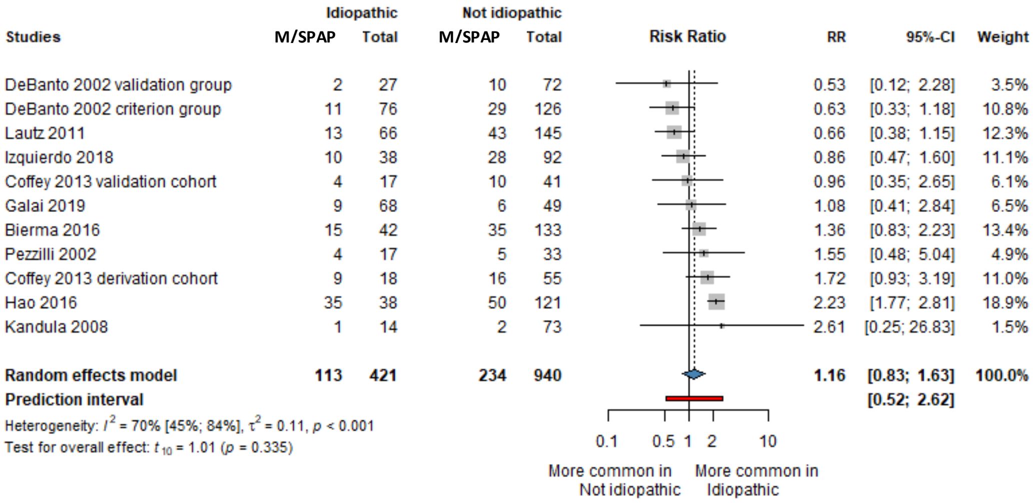

Supplementary Figure 15 Forest plot for idiopathic pediatric acute pancreatitis (M/SPAP: moderate or severe pediatric acute pancreatitis; RR: risk ratio; CI: confidence interval)

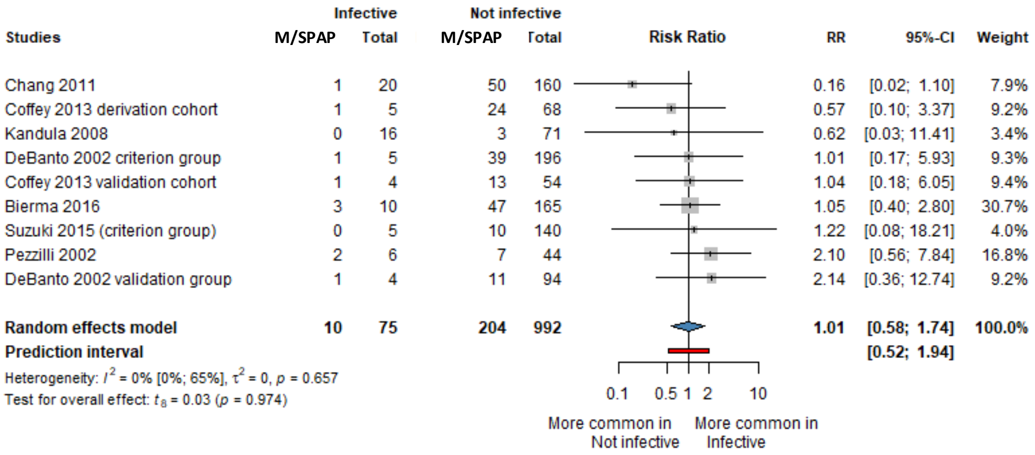

Supplementary Figure 16 Forest plot for infective pediatric acute pancreatitis (M/SPAP: moderate or severe pediatric acute pancreatitis; RR: risk ratio; CI: confidence interval)

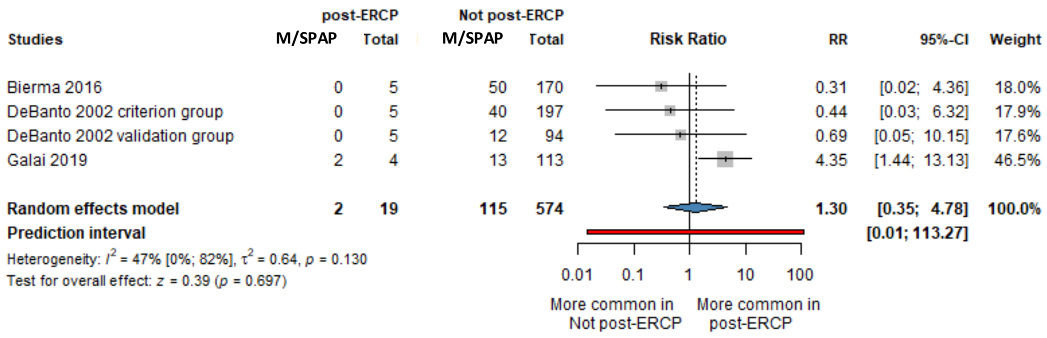

Supplementary Figure 17 Forest plot for post-ERCP pediatric acute pancreatitis (ERCP: endoscopic retrograde cholangio-pancreatography; M/SPAP: moderate or severe pediatric acute pancreatitis; RR: risk ratio; CI: confidence interval)

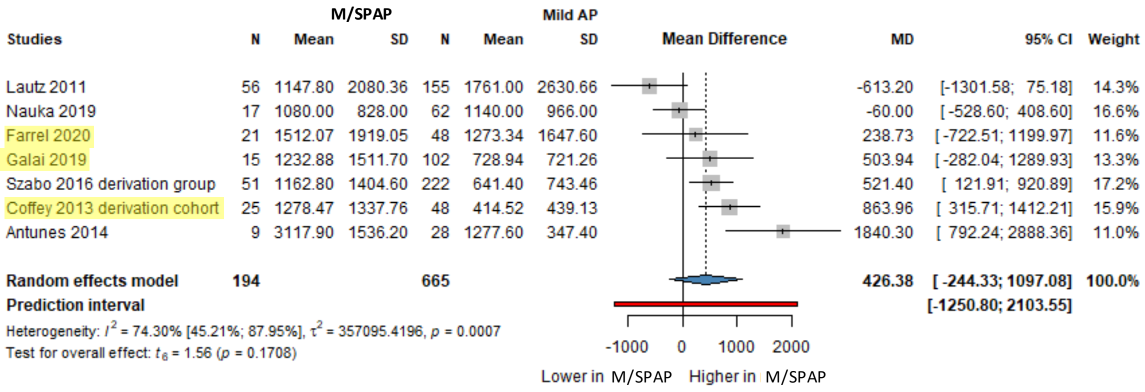

Supplementary Figure 18 Forest plot for lipase (U/L) levels in pediatric acute pancreatitis. A yellow background indicates that data had to be converted from medians with interquartile ranges to means with SD (AP: acute pancreatitis; M/SPAP: moderate or severe pediatric acute pancreatitis; N: patient number; SD: standard deviation; MD: mean difference; CI: confidence interval)

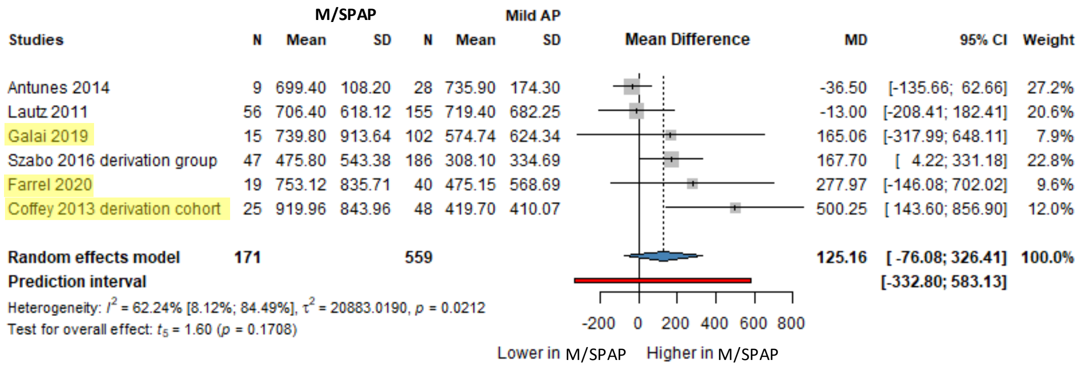

Supplementary Figure 19 Forest plot for amylase (U/L) levels in pediatric acute pancreatitis. A yellow background indicates that data had to be converted from medians with interquartile ranges to means with SD (AP: acute pancreatitis; M/SPAP: moderate or severe pediatric acute pancreatitis; N: patient number; SD: standard deviation; MD: mean difference; CI: confidence interval)

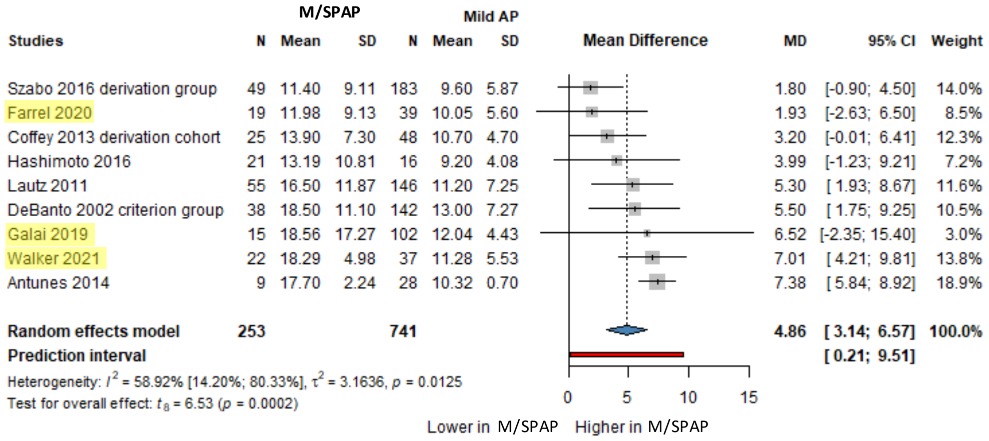

Supplementary Figure 20 Forest plot for white blood cell count (G/L) in pediatric acute pancreatitis. A yellow background indicates that data had to be converted from medians with interquartile ranges to means with SD (AP: acute pancreatitis; M/SPAP: moderate or severe pediatric acute pancreatitis; N: patient number; SD: standard deviation; MD: mean difference; CI: confidence interval)

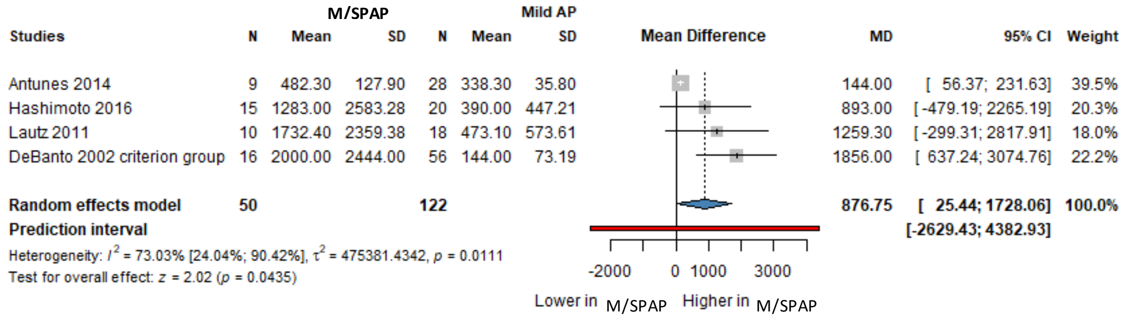

Supplementary Figure 21 Forest plot for lactate-dehydrogenase (U/L) levels in pediatric acute pancreatitis (AP: acute pancreatitis; M/SPAP: moderate or severe pediatric acute pancreatitis; N: patient number; SD: standard deviation; MD: mean difference; CI: confidence interval)

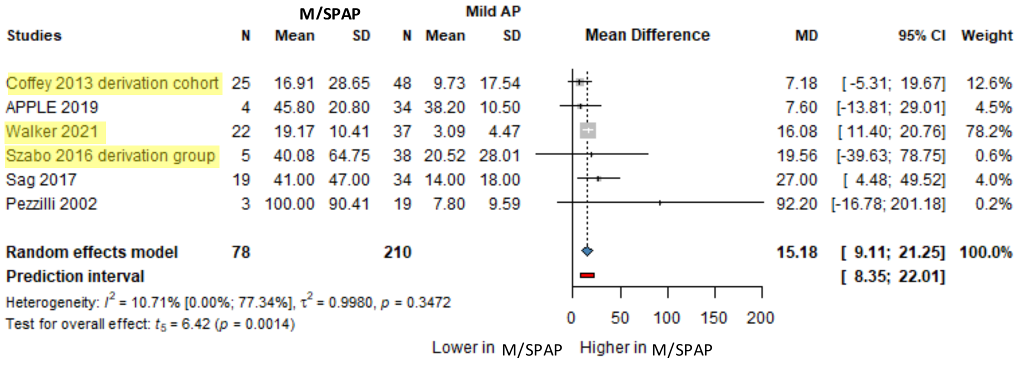

Supplementary Figure 22 Forest plot for C-reactive protein (mg/L) levels in pediatric acute pancreatitis. A yellow background indicates that data had to be converted from medians with

interquartile ranges to means with SD (AP: acute pancreatitis; M/SPAP: moderate or severe pediatric acute pancreatitis; N: patient number; SD: standard deviation; MD: mean difference; CI: confidence interval)

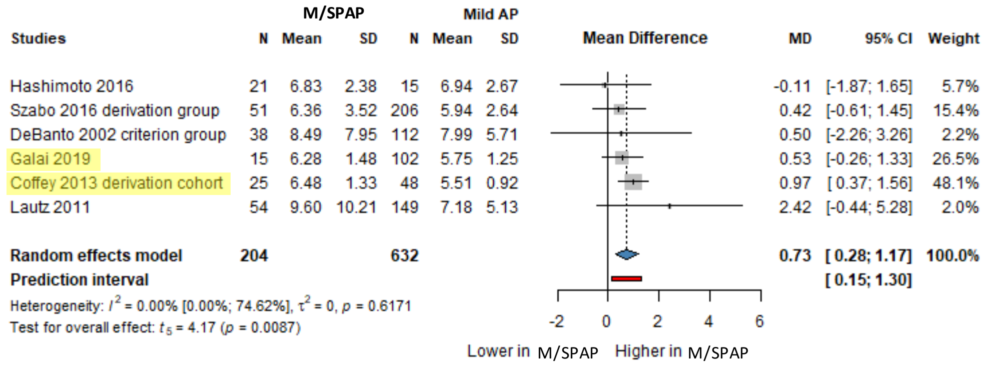

Supplementary Figure 23 Forest plot for glucose (mM/L) levels in pediatric acute pancreatitis. A yellow background indicates that data had to be converted from medians with interquartile ranges to means with SD (AP: acute pancreatitis; M/SPAP: moderate or severe pediatric acute pancreatitis; N: patient number; SD: standard deviation; MD: mean difference; CI: confidence interval)

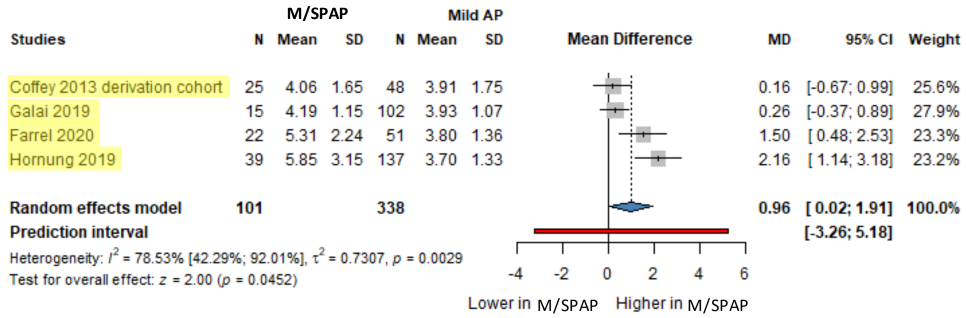

Supplementary Figure 24 Forest plot for blood urea nitrogen (BUN) (mM/L) levels in pediatric acute pancreatitis. A yellow background indicates that data had to be converted from medians with interquartile ranges to means with SD (AP: acute pancreatitis; M/SPAP: moderate or severe pediatric acute pancreatitis; N: patient number; SD: standard deviation; MD: mean difference; CI: confidence interval)

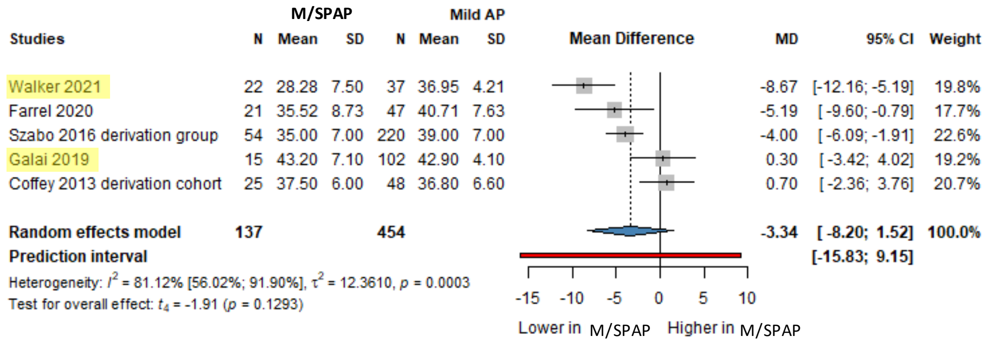

Supplementary Figure 25 Forest plot for albumin (g/L) levels in pediatric acute pancreatitis. A yellow background indicates that data had to be converted from medians with interquartile ranges to means with SD (AP: acute pancreatitis; M/SPAP: moderate or severe pediatric acute pancreatitis; N: patient number; SD: standard deviation; MD: mean difference; CI: confidence interval)

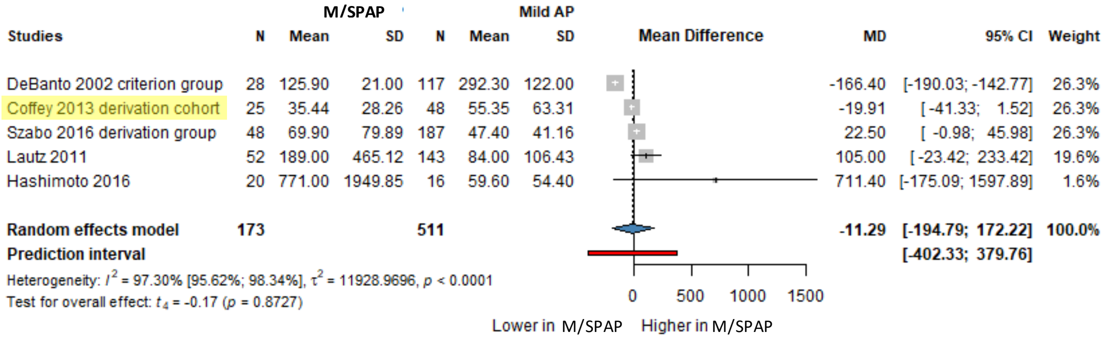

Supplementary Figure 26 Forest plot for aspartate transaminase (U/L) levels in pediatric acute pancreatitis. A yellow background indicates that data had to be converted from medians with interquartile ranges to means with SD (AP: acute pancreatitis; M/SPAP: moderate or severe pediatric acute pancreatitis; N: patient number; SD: standard deviation; MD: mean difference; CI: confidence interval)

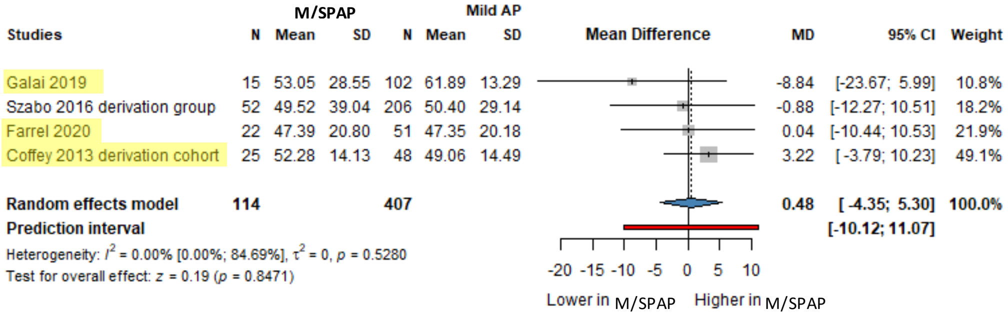

Supplementary Figure 27 Forest plot for creatinine (uM/L) levels in pediatric acute pancreatitis. A yellow background indicates that data had to be converted from medians with interquartile ranges to means with SD (AP: acute pancreatitis; M/SPAP: moderate or severe pediatric acute pancreatitis N: patient number; SD: standard deviation; MD: mean difference; CI: confidence interval)

Outcome	Study identifier	Study participation	Study attrition	Progn factor measurement	Outcome measurement	Study confounding	Statistical analysis reporting	Total assessment
**Age**	Bierma 2016	Low	Low	Low	Low	Low	Low	Low
	Chang 2011	Low	Low	Low	Low	Low	Low	Low
	Coffey 2013 derivation cohort	Low	Low	Low	Low	Low	Low	Low
	Coffey 2013 validation cohort	Low	Low	Low	Low	Low	Low	Low
	DeBanto 2002 criterion group	Low	Low	Low	Low	Low	Low	Low
	DeBanto 2002 validation group	Low	Low	Low	Low	Low	Low	Low
	Farrel 2020	Low	Low	Low	Low	Moderate	Low	Low
	Galai 2019	Low	Low	Low	Low	Low	Low	Low
	Hashimoto 2016	Low	Low	Low	Low	Moderate	Low	Low
	Izquierdo 2018	Low	Low	Low	Low	Low	Low	Low
	Lautz 2011	Moderate	Low	Low	Moderate	Low	Low	Moderate
	Li 2018	Low	Low	Low	Low	Low	Low	Low
	Nauka 2019	Low	Low	Low	Low	Low	Low	Low
	Suzuki 2015 (criterion group)	Moderate	Low	Low	Low	Low	Low	Low
	Szabo 2016 derivation group	Moderate	Low	Low	Low	Moderate	Low	Moderate
**Gender**	Bierma 2016	Low	Low	Low	Low	Low	Low	Low
	Coffey 2013 derivation cohort	Low	Low	Low	Low	Low	Low	Low
	Coffey 2013 validation cohort	Low	Low	Low	Low	Low	Low	Low
	DeBanto 2002 criterion group	Low	Low	Low	Low	Low	Low	Low
	DeBanto 2002 validation group	Low	Low	Low	Low	Low	Low	Low
	Farrel 2020	Low	Low	Low	Low	Moderate	Low	Low
	Galai 2019	Low	Low	Low	Low	Low	Low	Low
	Izquierdo 2018	Low	Low	Low	Low	Low	Low	Low
	Kandula 2008	Low	Moderate	Low	Low	Low	Low	Low
	Li 2018	Low	Low	Low	Low	Low	Low	Low
	Suzuki 2015 (criterion group)	Moderate	Low	Low	Low	Low	Low	Low
	Szabo 2016 derivation group	Moderate	Low	Low	Low	Moderate	Low	Moderate
**Previous AP**	Coffey 2013 derivation cohort	Low	Low	Moderate	Low	Low	Low	Low
	Coffey 2013 validation cohort	Low	Low	Moderate	Low	Low	Low	Low
	Galai 2019	Low	Low	Low	Low	Low	Low	Low
	Hao 2016	Moderate	Low	Low	Low	High	Low	High
	Mehta 2018	Low	Low	Moderate	Moderate	Low	Low	Moderate

Supplementary Figure 28 Results of the risk of bias assessment by the QUIPS tool for age, gender and previous acute pancreatitis (AP)

Outcome	Study identifier	Study participation	Study attrition	Progn factor measurement	Outcome measurement	Study confounding	Statistical analysis reporting	Total assessment
**Abdominal trauma**	Bierma 2016	Low	Low	Moderate	Low	Low	Low	Low
	Chang 2011	Low	Low	Moderate	Low	Low	Low	Low
	Coffey 2013 derivation cohort	Low	Low	Moderate	Low	Low	Low	Low
	Coffey 2013 validation cohort	Low	Low	Moderate	Low	Low	Low	Low
	DeBanto 2002 criterion group	Low	Low	Moderate	Low	Low	Low	Low
	DeBanto 2002 validation group	Low	Low	Moderate	Low	Low	Low	Low
	Galai 2019	Low	Low	Moderate	Low	Low	Low	Low
	Izquierdo 2018	Low	Low	Moderate	Low	Low	Low	Low
	Kandula 2008	Low	Moderate	Moderate	Low	Low	Low	Moderate
	Lautz 2011	Moderate	Low	Moderate	Moderate	Low	Low	Moderate
	Pezzilli 2002	Moderate	Low	Moderate	Low	Low	Low	Moderate
	Suzuki 2015 (criterion group)	Moderate	Low	Moderate	Low	Low	Low	Moderate
**Anatomical malformation**	Coffey 2013 derivation cohort	Low	Low	Moderate	Low	Low	Low	Low
	Coffey 2013 validation cohort	Low	Low	Moderate	Low	Low	Low	Low
	DeBanto 2002 criterion group	Low	Low	Moderate	Low	Low	Low	Low
	DeBanto 2002 validation group	Low	Low	Moderate	Low	Low	Low	Low
	Galai 2019	Low	Low	Moderate	Low	Low	Low	Low
	Izquierdo 2018	Low	Low	Moderate	Low	Low	Low	Low
	Kandula 2008	Low	Moderate	Moderate	Low	Low	Low	Moderate
	Lautz 2011	Moderate	Low	Moderate	Moderate	Low	Low	Moderate
	Pezzilli 2002	Moderate	Low	Moderate	Low	Low	Low	Moderate
	Suzuki 2015 (criterion group)	Moderate	Low	Moderate	Low	Low	Low	Moderate
**Drug-induced AP**	Bierma 2016	Low	Low	Moderate	Low	Low	Low	Low
	Chang 2011	Low	Low	Moderate	Low	Low	Low	Low
	Coffey 2013 derivation cohort	Low	Low	Moderate	Low	Low	Low	Low
	Coffey 2013 validation cohort	Low	Low	Moderate	Low	Low	Low	Low
	DeBanto 2002 criterion group	Low	Low	Moderate	Low	Low	Low	Low
	DeBanto 2002 validation group	Low	Low	Moderate	Low	Low	Low	Low
	Galai 2019	Low	Low	Moderate	Low	Low	Low	Low
	Izquierdo 2018	Low	Low	Moderate	Low	Low	Low	Low
	Kandula 2008	Low	Moderate	High	Low	Low	Low	High
	Lautz 2011	Moderate	Low	Moderate	Moderate	Low	Low	Moderate
	Pezzilli 2002	Moderate	Low	Moderate	Low	Low	Low	Moderate
	Suzuki 2015 (criterion group)	Moderate	Low	Moderate	Low	Low	Low	Moderate
**Biliary AP**	Bierma 2016	Low	Low	Moderate	Low	Low	Low	Low
	Chang 2011	Low	Low	Moderate	Low	Low	Low	Low
	Coffey 2013 derivation cohort	Low	Low	Moderate	Low	Low	Low	Low
	Coffey 2013 validation cohort	Low	Low	Moderate	Low	Low	Low	Low
	DeBanto 2002 criterion group	Low	Low	Moderate	Low	Low	Low	Low
	DeBanto 2002 validation group	Low	Low	Moderate	Low	Low	Low	Low
	Galai 2019	Low	Low	Moderate	Low	Low	Low	Low
	Hao 2016	Moderate	Low	Moderate	Low	High	Low	High
	Izquierdo 2018	Low	Low	Moderate	Low	Low	Low	Low
	Kandula 2008	Low	Moderate	Moderate	Low	Low	Low	Moderate
	Lautz 2011	Moderate	Low	Moderate	Moderate	Low	Low	Moderate
	Pezzilli 2002	Moderate	Low	Moderate	Low	Low	Low	Moderate
	Suzuki 2015 (criterion group)	Moderate	Low	Moderate	Low	Low	Low	Moderate
**Idiopathic AP**	Bierma 2016	Low	Low	Moderate	Low	Low	Low	Low
	Coffey 2013 derivation cohort	Low	Low	Moderate	Low	Low	Low	Low
	Coffey 2013 validation cohort	Low	Low	Moderate	Low	Low	Low	Low
	DeBanto 2002 criterion group	Low	Low	Moderate	Low	Low	Low	Low
	DeBanto 2002 validation group	Low	Low	Moderate	Low	Low	Low	Low
	Galai 2019	Low	Low	Moderate	Low	Low	Low	Low
	Hao 2016	Moderate	Low	Moderate	Low	High	Low	High
	Izquierdo 2018	Low	Low	Moderate	Low	Low	Low	Low
	Kandula 2008	Low	Moderate	Moderate	Low	Low	Low	Moderate
	Lautz 2011	Moderate	Low	Moderate	Moderate	Low	Low	Moderate
	Pezzilli 2002	Moderate	Low	Moderate	Low	Low	Low	Moderate
**Infective etiology**	Bierma 2016	Low	Low	Moderate	Low	Low	Low	Low
	Chang 2011	Low	Low	Moderate	Low	Low	Low	Low
	Coffey 2013 derivation cohort	Low	Low	Moderate	Low	Low	Low	Low
	Coffey 2013 validation cohort	Low	Low	Moderate	Low	Low	Low	Low
	DeBanto 2002 criterion group	Low	Low	Moderate	Low	Low	Low	Low
	DeBanto 2002 validation group	Low	Low	Moderate	Low	Low	Low	Low
	Kandula 2008	Low	Moderate	Moderate	Low	Low	Low	Moderate
	Pezzilli 2002	Moderate	Low	Moderate	Low	Low	Low	Moderate
	Suzuki 2015 (criterion group)	Moderate	Low	Moderate	Low	Low	Low	Moderate
**Post-ERCP AP**	Bierma 2016	Low	Low	Moderate	Low	Low	Low	Low
	DeBanto 2002 criterion group	Low	Low	Moderate	Low	Low	Low	Low
	DeBanto 2002 validation group	Low	Low	Moderate	Low	Low	Low	Low
	Galai 2019	Low	Low	Moderate	Low	Low	Low	Low

Supplementary Figure 29 Results of the risk of bias assessment by the QUIPS tool for etiological factors (AP: acute pancreatitis)

Outcome	Study identifier	Study participation	Study attrition	Progn factor measurement	Outcome measurement	Study confounding	Statistical analysis reporting	Total assessment
Lipase	Antunes 2014	Moderate	Low	Low	Moderate	Low	Low	Moderate
	Coffey 2013 derivation cohort	Low	Low	Low	Low	Low	Low	Low
	Farrel 2020	Low	Low	Low	Low	Moderate	Low	Low
	Galai 2019	Low	Low	Low	Low	Low	Low	Low
	Lautz 2011	Moderate	Low	Low	Moderate	Low	Low	Moderate
	Nauka 2019	Low	Low	Low	Low	Low	Low	Low
	Szabo 2016 derivation group	Moderate	Low	Low	Low	Moderate	Low	Moderate
Amylase	Antunes 2014	Moderate	Low	Low	Moderate	Low	Low	Moderate
	Coffey 2013 derivation cohort	Low	Low	Low	Low	Low	Low	Low
	Farrel 2020	Low	Low	Moderate	Low	Moderate	Low	Moderate
	Galai 2019	Low	Low	Low	Low	Low	Low	Low
	Lautz 2011	Moderate	Low	Low	Moderate	Low	Low	Moderate
	Szabo 2016 derivation group	Moderate	Low	Moderate	Low	Moderate	Low	Moderate
White blood cell count	Antunes 2014	Moderate	Low	Low	Moderate	Low	Low	Moderate
	Coffey 2013 derivation cohort	Low	Low	Low	Low	Low	Low	Low
	DeBanto 2002 criterion group	Low	Low	Low	Low	Low	Low	Low
	Farrel 2020	Low	Low	Moderate	Low	Moderate	Low	Moderate
	Galai 2019	Low	Low	Low	Low	Low	Low	Low
	Hashimoto 2016	Low	Low	Low	Low	Moderate	Low	Low
	Lautz 2011	Moderate	Low	Low	Moderate	Low	Low	Moderate
	Szabo 2016 derivation group	Moderate	Low	Moderate	Low	Moderate	Low	Moderate
	Walker 2021	Low	Low	Low	Low	Low	Low	Low
Lactate-dehydro-genase	Antunes 2014	Moderate	Low	Low	Moderate	Low	Low	Moderate
	DeBanto 2002 criterion group	Low	Low	High	Low	Low	Low	Moderate
	Hashimoto 2016	Low	Low	Low	Low	Moderate	Low	Low
	Lautz 2011	Moderate	Low	High	Moderate	Low	Low	High
C-reactive protein	Coffey 2013 derivation cohort	Low	Low	Low	Low	Low	Low	Low
	APPLE 2019	Low	Low	Low	Low	Moderate	Low	Low
	Pezzilli 2002	Moderate	Low	High	Low	Low	Low	High
	Sag 2017	Low	Low	Moderate	Low	Low	Low	Low
	Szabo 2016 derivation group	Moderate	Low	High	Low	Moderate	Low	High
	Walker 2021	Low	Low	Low	Low	Low	Low	Low
Glucose	Coffey 2013 derivation cohort	Low	Low	Moderate	Low	Low	Low	Low
	DeBanto 2002 criterion group	Low	Low	Moderate	Low	Low	Low	Low
	Galai 2019	Low	Low	Moderate	Low	Low	Low	Low
	Hashimoto 2016	Low	Low	Low	Low	Moderate	Low	Low
	Lautz 2011	Moderate	Low	Low	Moderate	Low	Low	Moderate
	Szabo 2016 derivation group	Moderate	Low	Moderate	Low	Moderate	Low	Moderate
Blood urea nitrogen	Coffey 2013 derivation cohort	Low	Low	Moderate	Low	Low	Low	Low
	Farrel 2020	Low	Low	Low	Low	Moderate	Low	Low
	Galai 2019	Low	Low	Moderate	Low	Low	Low	Low
	Hornung 2019	Moderate	Low	Low	Low	Moderate	Low	Moderate
Albumin	Coffey 2013 derivation cohort	Low	Low	Moderate	Low	Low	Low	Low
	Farrel 2020	Low	Low	Moderate	Low	Moderate	Low	Moderate
	Galai 2019	Low	Low	Moderate	Low	Low	Low	Low
	Szabo 2016 derivation group	Moderate	Low	Low	Low	Moderate	Low	Moderate
	Walker 2021	Low	Low	Moderate	Low	Low	Low	Low
Aspartate transaminase	Coffey 2013 derivation cohort	Low	Low	Moderate	Low	Low	Low	Low
	DeBanto 2002 criterion group	Low	Low	Moderate	Low	Low	Low	Low
	Hashimoto 2016	Low	Low	Low	Low	Moderate	Low	Low
	Lautz 2011	Moderate	Low	Low	Moderate	Low	Low	Moderate
	Szabo 2016 derivation group	Moderate	Low	Moderate	Low	Moderate	Low	Moderate
Creatinine	Coffey 2013 derivation cohort	Low	Low	Moderate	Low	Low	Low	Low
	Farrel 2020	Low	Low	Low	Low	Moderate	Low	Low
	Galai 2019	Low	Low	Moderate	Low	Low	Low	Low
	Szabo 2016 derivation group	Moderate	Low	Moderate	Low	Moderate	Low	Moderate

Supplementary Figure 30 Results of the risk of bias assessment by the QUIPS tool for on-admission laboratory parameters

Study identifier	Citation(s) of report(s)	Study identifier	Citation(s) of report(s)
Abu-El-Haija 2020	(1, 2)	Izquierdo 2018	(3)
Antunes 2014	(4, 5)	Kandula 2008	(6)
Berney 1996	(7)	Kaur 2018	(8)
Bierma 2016	(9, 10)	APPLE 2019	(11-16)
Birimberg-Schwartz 2021	(17)	Lautz 2011	(18, 19)
Boskovic 2014	(20)	Li 2018	(21)
Chang 2011	(22)	Mehta 2018	(23)
Coffey 2013 derivation cohort	(24-26)	Nauka 2019	(27, 28)
Coffey 2013 validation cohort		Orkin 2017	(29)
DeBanto 2002 criterion group	(30)	Parian 2019	(31)
DeBanto 2002 validation group		Pezzili 2002	(32)
Fabre 2012	(33, 34)	Sag 2017	(35)
Farrel 2020	(36-40)	Sánchez-Ramírez 2007	(41)
Farrel 2021 derivation cohort	(42, 43)	Suzuki 2015 criterion group	(44, 45)
Farrel 2021 validation cohort		Suzuki 2017 validation group	(46)
Fonseca Sepúlveda 2019	(47)	Szabo 2016 derivation group	(48, 49)
Galai 2019	(50, 51)	Szabo 2016 validation group	
Guerrero-Lozano 2017	(52)	Thavamani 2021	(53-55)
Hao 2016	(56)	Vitale 2019	(57, 58)
Hashimoto 2016	(59)	Walker 2021	(60, 61)
Hornung 2019	(62)	Wetherill 2013	(63-65)
Izquierdo 2018	(66, 67)	Zheng 2018	(68)

Supplementary Table 1: Citations of study reports. *Study identifiers are presented as in Table 1 and citations of corresponding reports are listed.*

REFERENCES

1. Abu-El-Haija M, Hornung L, Lin TK, Nathan JD, Thompson T, Vitale DS, et al. DRUG-INDUCED PANCREATITIS IS THE LEADING RISK FACTOR FOR FIRST ATTACK OF ACUTE PANCREATITIS IN CHILDREN. Gastroenterology. 2020;158(6):S-589-S-90.
2. Abu-El-Haija M, Hornung L, Lin TK, Nathan JD, Thompson T, Vitale DS, et al. Drug induced pancreatitis is the leading known cause of first attack acute pancreatitis in children. Pancreatology. 2020;20(6):1103-8.
3. Izquierdo YE, Fonseca EV, Moreno L, Montoya RD, Guerrero R. Multivariate Model for the Prediction of Severity of Acute Pancreatitis in Children. J Pediatr Gastroenterol Nutr. 2018;66(6):949-52.
4. Antunes H, Nascimento J, Mesquita A, Correia-Pinto J. Acute pancreatitis in children: a tertiary hospital report. Scand J Gastroenterol. 2014;49(5):642-7.
5. Ferreira C, Estrada A, Correia-Pinto J, Antunes H. Acute pancreatitis in children: Spectrum of disease and predictors of severity. Journal of Pediatric Gastroenterology and Nutrition. 2016;63:S410.
6. Kandula L, Lowe ME. Etiology and outcome of acute pancreatitis in infants and toddlers. J Pediatr. 2008;152(1):106-10, 10.e1.
7. Berney T, Belli D, Bugmann P, Beghetti M, Morel P, LeCoultre C. Influence of severe underlying pathology and hypovolemic shock on the development of acute pancreatitis in children. J Pediatr Surg. 1996;31(9):1256-61.
8. Kaur J, Wadhwa N, Jeena S. Classifying pediatric acute pancreatitis into mild, moderately severe and severe! Journal of Pediatric Gastroenterology and Nutrition. 2018;66:242.
9. Bierma M, Coffey MJ, Nightingale S, Van Rheenen P, Ooi CY. Predicting severe acute pancreatitis in children based on serum lipase and calcium: A multicentre retrospective cohort study. Gastroenterology. 2016;150(4):S711.
10. Bierma MJ, Coffey MJ, Nightingale S, van Rheenen PF, Ooi CY. Predicting severe acute pancreatitis in children based on serum lipase and calcium: A multicentre retrospective cohort study. Pancreatology. 2016;16(4):529-34.
11. Lásztity N, Mosztbacher D, Juhász FM, Kaán K, Tokodi I, Tészás A, et al. Clinical signs of severity and therapeutic intervention in pediatric acute pancreatitis-data from APPLE multicentre, observational clinical trial. Journal of Pediatric Gastroenterology and Nutrition. 2019;68:212.
12. Lasztity N, Mosztbacher D, Toth AZ, Demcsak A, Tokodi I, Czelecz J, et al. Clinical signs of severity and therapeutic intervention in pediatric acute pancreatitis e prospective multicenter nation-wide cohort. Pancreatology. 2018;18(4):S20-S1.
13. Martonosi AR, Mosztbacher D, Juhász MF, Tokodi I, Tészás A, Gárdos L, et al. Diabetic ketoacidosis worsen the outcome of AP in childhood – preliminary results of APPLE (Analysis of

Pediatric Pancreatitis) prospectice, multicentric, observational clinical trial. Pancreatology. 2019;19:S90-S1.
14. Parniczky A, Lásztity N, Mosztbacher D, Tóth A, Szucs D, Vass N, et al. Pediatric pancreatitis. Multicentre prospective data collection and analysis by the hungarian pancreatic study group. Pancreatology. 2015;15(3):S65.
15. Párniczky A, Németh BC, Mosztbacher D, Szentesi A, Abu-el Haija M, Pienar C, et al. Explore the childhood onset pancreatitis with the support of APPLE (analysis of pediatric pancreatitis) multicenter, observational, clinical trial. Journal of Pediatric Gastroenterology and Nutrition. 2019;68:223-4.
16. Párniczky A, Németh BC, Mosztbacher D, Tóth AZ, Lásztity N, Abu-El-Haija M, et al. Explore the childhood onset pancreatitis with the support of APPLE (Analysis of Pediatric Pancreatitis) multicenter, observational, clinical trial. Pancreas. 2019;48(10):1506.
17. Birimberg-Schwartz L, Rajiwate S, Dupuis A, Gonska T. Pediatric Acute Pancreatitis: Changes in Management and Disease Outcomes Over 16 Years. Pancreas. 2021;50(3):341-6.
18. Lautz TB, Chin AC, Radhakrishnan J. Acute pancreatitis in children: spectrum of disease and predictors of severity. J Pediatr Surg. 2011;46(6):1144-9.
19. Lautz TB, Turkel G, Radhakrishnan J, Wyers M, Chin AC. Utility of the computed tomography severity index (Balthazar score) in children with acute pancreatitis. J Pediatr Surg. 2012;47(6):1185-91.
20. Boskovic A, Pasic S, Soldatovic I, Milinic N, Stankovic I. The role of D-dimer in prediction of the course and outcome in pediatric acute pancreatitis. Pancreatology. 2014;14(5):330-4.
21. Li W, Luo S, Zhu Y, Shu M, Wen Y, Wang Z, et al. Concordance of the Balthazar Grade and the Revised Atlanta Classification: Proposing a Modified Balthazar Grade to Predict the Severity of Acute Pancreatitis in Pediatric Population. Pancreas. 2018;47(10):1312-6.
22. Chang YJ, Chao HC, Kong MS, Hsia SH, Lai MW, Yan DC. Acute pancreatitis in children. Acta Paediatr. 2011;100(5):740-4.
23. Mehta M, Baldwin C, Sathe M, Troendle D. Severe pancreatitis is rare in pediatric patients: Results from a retrospective review. Journal of Pediatric Gastroenterology and Nutrition. 2018;67:S255.
24. Coffey MJ, Ooi CY. Can serum pancreatic enzyme levels predict severity of acute pediatric pancreatitis? Pancreatology. 2011;11(6):570-1.
25. Coffey MJ, Nightingale S, Ooi CY. Early prediction of severity in acute pediatric pancreatitis. Gastroenterology. 2012;142(5):S848.
26. Coffey MJ, Nightingale S, Ooi CY. Serum lipase as an early predictor of severity in pediatric acute pancreatitis. J Pediatr Gastroenterol Nutr. 2013;56(6):602-8.
27. Nauka PC, Dolinger MT, Kohn N, Miller JM, Bitton S, Weinstein T, et al. Sirs is a Prognostic Indicator for Pediatric Severe Acute Pancreatitis: A Retrospective Analysis. Gastroenterology. 2018;154(6):S-710-S-1.
28. Nauka PC, Weinstein TA, Dolinger MT, Miller JM, Kohn N, Bitton S, et al. Validation of Lipase and Systemic Inflammatory Response Syndrome as Prognostic Indicators in Pediatric Acute Pancreatitis: A Retrospective Analysis. J Pediatr Gastroenterol Nutr. 2019;68(3):389-93.
29. Orkin SH, Lin TK, Fei L, Nathan JD, Thompson T, Abu-El-Haija M. Serum amylase and lipase patterns in pediatric acute pancreatitis diagnosis: Is serum amylase really needed? Gastroenterology. 2017;152(5):S435.
30. DeBanto JR, Goday PS, Pedroso MR, Iftikhar R, Fazel A, Nayyar S, et al. Acute pancreatitis in children. Am J Gastroenterol. 2002;97(7):1726-31.
31. Parian E, Tan M, Urtula R, Nolasco ME. Clinical profile and outcome of acute pancreatitis in children admitted in philippine children's medical center. Gut. 2019;68:A15.
32. Pezzilli R, Morselli-Labate AM, Castellano E, Barbera C, Corrao S, Di Prima L, et al. Acute pancreatitis in children. An Italian multicentre study. Dig Liver Dis. 2002;34(5):343-8.
33. Fabre A, Petit P, Gaudart J, Mas E, Vial J, Olives J, et al. Assessment of the interest of gravity score in acute pancreatitis of children. Journal of Pediatric Gastroenterology and Nutrition. 2009;48:E57-E8.
34. Fabre A, Petit P, Gaudart J, Mas E, Vial J, Olives JP, et al. Severity scores in children with acute pancreatitis. J Pediatr Gastroenterol Nutr. 2012;55(3):266-7.

35. Sag E, Akbulut UE, Comert HSY, Ozdogan EB, Karahan SC, Cakir M. Acute pancreatitis in children: Single center experince over 10-year period. Journal of Pediatric Gastroenterology and Nutrition. 2017;64:251.
36. Farrell PR, Hornung L, Farmer P, DesPain AW, Kim E, Pearman R, et al. Who's at Risk? A Prognostic Model for Severity Prediction in Pediatric Acute Pancreatitis. J Pediatr Gastroenterol Nutr. 2020;71(4):536-42.
37. Farrell P, Hornung L, Farmer P, DesPain A, Kim E, Pearman R, et al. Who's at risk? A prognostic model for severity prediction in pediatric acute pancreatitis. Journal of Pediatric Gastroenterology and Nutrition. 2019;69.
38. Farrell P, Hornung L, Farmer P, Serrette A, Lin T, Nathan J, et al. Bun change from admission and levels at 24-48 hours are significant predictors of severity in pediatric acute pancreatitis. Journal of Pediatric Gastroenterology and Nutrition. 2019;69.
39. Farrell PR, Hornung L, Farmer PF, DesPain AW, Kim E, Pearman R, et al. VALIDATION AND OPTIMIZATION OF AN EARLY PREDICTOR MODEL FOR PEDIATRIC ACUTE PANCREATITIS. Gastroenterology. 2019;156(6):S-342.
40. Farrell P, Serrette A, Farmer P. Bun levels: A marker for severity in pediatric acute pancreatitis? Journal of Pediatric Gastroenterology and Nutrition. 2017;65:S329-S30.
41. Sánchez-Ramírez CA, Larrosa-Haro A, Flores-Martínez S, Sánchez-Corona J, Villa-Gómez A, Macías-Rosales R. Acute and recurrent pancreatitis in children: etiological factors. Acta Paediatr. 2007;96(4):534-7.
42. Farrell PR, Jones EK, Hornung L, Thompson T, Patel J, Lin TK, et al. Cytokine Profile Elevations on Admission Can Determine Risks of Severe Acute Pancreatitis in Children. J Pediatr. 2021;238:33-41.e4.
43. Farrell PR, Jones EK, Hornung L, Thompson T, Patel J, Lin TK, et al. USE OF NOVEL CYTOPLEX ASSAY TO DETERMINE PREDICTORS OF SEVERITY IN PEDIATRIC ACUTE PANCREATITIS. Gastroenterology. 2020;158(6):S-607.
44. Suzuki M, Saito N, Naritaka N, Nakano S, Minowa K, Honda Y, et al. Scoring system for the prediction of severe acute pancreatitis in children. Pediatr Int. 2015;57(1):113-8.
45. Suzuki M, Fujii T, Takahiro K, Ohtsuka Y, Nagata S, Shimizu T. Scoring system for the severity of acute pancreatitis in children. Pancreas. 2008;37(2):222-3.
46. Suzuki M, Saito N, Minowa K, Kagimoto S, Shimizu T. Validation of severity assessment for acute pancreatitis in children. Pediatr Int. 2017;59(10):1127-8.
47. Fonseca Sepúlveda EV, Guerrero-Lozano R. Acute pancreatitis and recurrent acute pancreatitis: an exploration of clinical and etiologic factors and outcomes. J Pediatr (Rio J). 2019;95(6):713-9.
48. Szabo FK, Hornung L, Oparaji JA, Alhosh R, Husain SZ, Liu QY, et al. A prognostic tool to predict severe acute pancreatitis in pediatrics. Pancreatology. 2016;16(3):358-64.
49. Szabo FK, Hornung L, Fei L, Palermo JJ, Lin TK, Nathan JD, et al. Early predictors of severe pancreatitis in children. Gastroenterology. 2015;148(4):S682.
50. Galai T, Cohen S, Yerushalmy-Feler A, Weintraub Y, Moran-Lev H, Amir AZ. Young Age Predicts Acute Pancreatitis Severity in Children. J Pediatr Gastroenterol Nutr. 2019;68(5):720-6.
51. Galai T, Cohen S, Feler AY, Weintraub Y, Weiner D, Amir A. Amylase, lipase and white blood cell count at presentation are predictors of severity of acute pancreatitis in children. Journal of Pediatric Gastroenterology and Nutrition. 2017;64:246.
52. Guerrero-Lozano R, Izquierdo Y, Fonseca V, Moreno LA, Montoya R. Utility of tomographic classifications in predicting unfavorable outcomes in children with acute pancreatitis. Journal of Pediatric Gastroenterology and Nutrition. 2017;65:S333-S4.
53. Thavamani A, Umapathi KK, Sferra TJ, Sankararaman S. Undernutrition and obesity are associated with adverse clinical outcomes in hospitalized children and adolescents with acute pancreatitis. Nutrients. 2021;13(1):1-10.
54. Thavamani A, Umapathi K, Sferra T, Sankararaman S. Nutritional status adversely predicts the severity of acute pancreatitis in hospitalized pediatric patients. Journal of Parenteral and Enteral Nutrition. 2021;45(SUPPL 1):S184-S7.
55. Thavamani A, Umapathi KK, Roy A, Krishna SG. The increasing prevalence and adverse impact of morbid obesity in pediatric acute pancreatitis. Pediatr Obes. 2020;15(8):e12643.

56. Hao F, Guo H, Luo Q, Guo C. Disease progression of acute pancreatitis in pediatric patients. J Surg Res. 2016;202(2):422-7.
57. Vitale DS, Hornung L, Lin TK, Nathan JD, Prasad S, Thompson T, et al. Blood Urea Nitrogen Elevation Is a Marker for Pediatric Severe Acute Pancreatitis. Pancreas. 2019;48(3):363-6.
58. Vitale D, Hornung L, Lin T, Thompson T, Nathan J, Abu-El-Haija M. Early predictors of severe acute pancreatitis in a prospective pediatric cohort. Journal of Pediatric Gastroenterology and Nutrition. 2017;65:S212-S3.
59. Hashimoto N, Yotani N, Michihata N, Tang J, Sakai H, Ishiguro A. Efficacy of pediatric acute pancreatitis scores at a Japanese tertiary center. Pediatr Int. 2016;58(3):224-8.
60. Walker H, Melling J, Jones M, Melling C. C-reactive protein accurately predicts severity of acute pancreatitis in children. British Journal of Surgery. 2021;108(SUPPL 6):vi181.
61. Walker H, Melling J, Jones M, Melling CV. C-reactive protein accurately predicts severity of acute pancreatitis in children. J Pediatr Surg. 2021.
62. Hornung LN, Farrell PR, Farmer P, Serrette A, Lin TK, Nathan JD, et al. BUN change from admission and levels at 24-48 hours are significant predictors of severity in pediatric acute pancreatitis. Pancreas. 2019;48(10):1445.
63. Wetherill C, Melling J, Jones M. C-reactive protein accurately predicts severity of acute pancreatitis in children. Pancreatology. 2013;13(2):e84.
64. Wetherill C, Melling J, Jones M. 48 hour C-reactive protein accurately predicts severity of acute pancreatitis in children. Pancreatology. 2013;13(1):e6.
65. Wetherill C, Melling J, Jones M. C-reactive protein accurately predicts severity of acute pancreatitis in children. Pancreas. 2012;41(8):1412-3.
66. Izquierdo YE, Fonseca EV, Moreno L, Montoya RD, Guerrero Lozano R. Utility of CT classifications to predict unfavorable outcomes in children with acute pancreatitis. Pediatr Radiol. 2018;48(7):954-61.
67. Izquierdo Y, Fonseca E, Moreno LA, Montoya R, Guerrero R. Utility of tomographic classifications in predicting unfavorable outcomes in children with acute pancreatitis. Pancreatology. 2017;17(4):S44.
68. Zheng W, Zhang L, Long G, Chen B, Shu X, Jiang M. Amalgamation of systemic inflammatory response syndrome score with C-reactive protein level in evaluating acute pancreatitis severity in children. Scand J Gastroenterol. 2018;53(6):755-9.
